# Effects of Temperature, Operation Mode, and Steam Concentration on Alkali Release in Chemical Looping Conversion of Biomass—Experimental Investigation in a 10 kW_th_ Pilot

**DOI:** 10.1021/acs.energyfuels.1c04353

**Published:** 2022-03-09

**Authors:** Ivan Gogolev, Amir H. Soleimanisalim, Daofeng Mei, Anders Lyngfelt

**Affiliations:** Chalmers University of Technology, Hörsalsvägen 7, Göteborg SE-412 96, Sweden

## Abstract

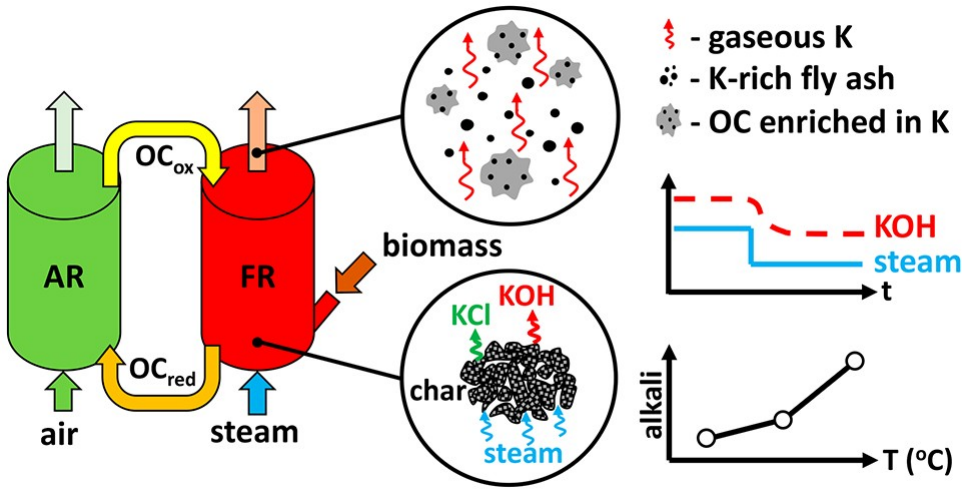

Alkali release was studied in a 10 kW_th_ chemical looping pilot operated with a Linz–Donawitz (LD) slag oxygen carrier (OC) and three biomass fuels. Experiments were performed at three temperatures and in three operation modes: chemical looping combustion (CLC), chemical looping gasification (CLG), and oxygen-carrier-aided combustion (OCAC). Gas-phase alkali release was measured with a surface ionization detector (SID). Fuel reactor (FR) gas-phase alkali emissions increased with the temperature. This occurred as a result of increased evaporation of KCl and enhanced decomposition of alkali salts during char conversion. Air reactor (AR) alkali emissions were lower than in the FR and independent of the operating temperature. In comparison of operating modes, CLC and CLG modes resulted in similar gas-phase alkali emissions due to the similar extent of char conversion. In contrast, operation of the reactor system in OCAC mode resulted in significantly lower levels of gas-phase alkalis. The difference in alkali emission was attributed to the steam-rich atmosphere of CLC. The effect of steam was further investigated in CLC and OCAC tests. Lowering steam concentrations in CLC operation resulted in lower gas-phase alkali emissions, while introducing steam to the FR during OCAC operation resulted in higher alkali emissions. It was concluded that steam likely enhances gas-phase K release through a reaction of K_2_CO_3_ within the fuel char with steam to produce KOH(g). Solid sampling and analysis for K content was used along with SID measurements to develop a K mass balance for the reactor system. Mass balance results for the straw pellet fuel tests showed that LD slag OC absorbs approximately 15–51% of fuel K, 2.2% of fuel K is released to the gas phase, and up to 3.4% of fuel K is captured in the AR fly ash. The residual 40–80% of fuel K was determined to leave the FR as K-rich fly ash.

## 1 Introduction

Chemical looping combustion (CLC) and chemical looping gasification (CLG) of biomass fuels are promising new technologies for bioenergy with carbon capture and storage (BECCS). The CLC and CLG process scheme relies on cyclical redox of a solid oxygen carrier (OC) material, which is continuously circulated between two reactors that are interconnected for solid circulation but do not allow for the gases from the two reactors to mix. The circulating OC material is oxidized by air in the air reactor (AR) and is reduced by fuel in the fuel reactor (FR). Because the OC delivers oxygen in solid form from the AR to the FR, nitrogen from AR air is kept from entering the FR product gas stream, making carbon capture inherent to the chemical looping operating principle. Details on the principle of CLC and CLG as well as progress in the chemical looping suite of technologies have been described in detail in a number of publications.^1−7^ Although CLC and CLG systems require the use of an oxygen carrier as the bed material and require a more complex dual reactor arrangement, the inherent carbon capture of these systems comes at much lower cost and energy penalties when compared to conventional post-combustion carbon capture or oxy-fuel combustion technologies.^8−10^

The inherently different operation principle of CLC and CLG poses a lot of unknowns and challenges for effective conversion of biomass fuels. A key challenge for effectively using biomass in chemical looping conversion lies in understanding and managing the release of alkali compounds during biomass conversion. In conventional biomass combustion and gasification, alkali compounds are released during fuel conversion and have significant implications on system operation. In biomass boilers, alkali compounds are known to cause severe fouling and corrosion of heat exchange equipment. In fact, biomass boilers operated with high-alkali biomass fuels typically limit the steam temperature to values below 500 °C to avoid alkali-related corrosion and fouling issues. This, however, greatly limits the boiler thermodynamic efficiency. In fluidized bed biomass boilers, alkalis that are retained in the bed are known to cause bed material agglomeration that can result in operational upsets and unplanned boiler shutdowns.^11,12^ The release of alkali compounds in the conventional combustion, gasification, and pyrolysis context has been studied in full scale in biomass boilers^12−14^ and gasifiers,^15−17^ in pilot-scale reactors,^12,18^ at the lab scale,^18−21^ and through modeling work.^22,23^ In CLC, the implications of alkali release from biomass fuel conversion are not yet well-explored. The majority of published research on the effects of alkalis in CLC has been carried out in lab-scale investigations and has been primarily focused on the interaction of alkali-containing fuel ash components with the oxygen carriers.^24−27^ Two pilot-scale investigations in continuous CLC systems have looked at effects of alkalis on oxygen carriers,^28,29^ but only a few studies have attempted to directly measure and determine alkali release to the gas phase and alkali retention in the bed material.^30−32^

Initial campaigns measuring alkali emissions in pilot CLC operation^30−32^ reported the first FR and AR alkali release figures for several biomass fuels, establishing that overall alkali emissions correlate with the alkali content of the fuel and that FR alkali emissions are higher than AR alkali emissions in most cases. It was also found that AR alkali emissions can be significant and can occur as a result of char carryover from the FR to the AR. The effect of the ilmenite and braunite oxygen carriers used in the initial campaigns was found to be significant, with >92% of fuel alkali estimated to be absorbed by the oxygen carrier or to remain in condensed form within the CLC systems. In the latest study, a comparison of CLC operation to operation of the same pilot system as a circulating fluidized bed boiler with oxygen carrier bed material, the so-called oxygen-carrier-aided combustion (OCAC) mode, showed that OCAC operation results in significantly lower alkali emissions.^32^ The higher emissions in CLC operation were suspected to occur as a result of the steam-rich environment in CLC operation.

The purpose of the current study was to further expand the understanding of alkali release and retention behavior in continuous chemical looping conversion of biomass. The current experiments aimed at determining how alkali release in a 10 kW_th_ CLC pilot using Linz–Donawitz (LD) slag as the oxygen carrier is influenced by the reactor temperature, reactor mode of operation, and fluidizing steam concentration.

## 2 Experimental Section

CLC and CLG experiments were conducted in a 10 kW_th_ CLC pilot system located at Chalmers University of Technology. The pilot was operated with the LD slag oxygen carrier, a waste slag material from a steel-making process. Three different biomass fuels of varying alkali content were tested: black pellets (BP), pine forest residue (PFR), and straw pellets (SP). The test campaign spanned 6 days of fueled operation. The main aim of the experiments was to evaluate how process temperature and type of operation affect AR and FR alkali emissions. CLC and CLG tests were conducted at 870, 920, and 970 °C for BP and PFR fuels and 870 and 970 °C for the SP fuel. Additional tests were also conducted in OCAC mode. OCAC tests were conducted at 920 °C for BP fuel and 970 °C for PFR and SP fuels. Several shorter tests were also conducted to test the effect of the steam concentration on alkali release during CLC and OCAC operation. Details on the experimental setup and experimental procedures are detailed in the following sections.

### 2.1 Pilot Reactor System

**Figure 1:**
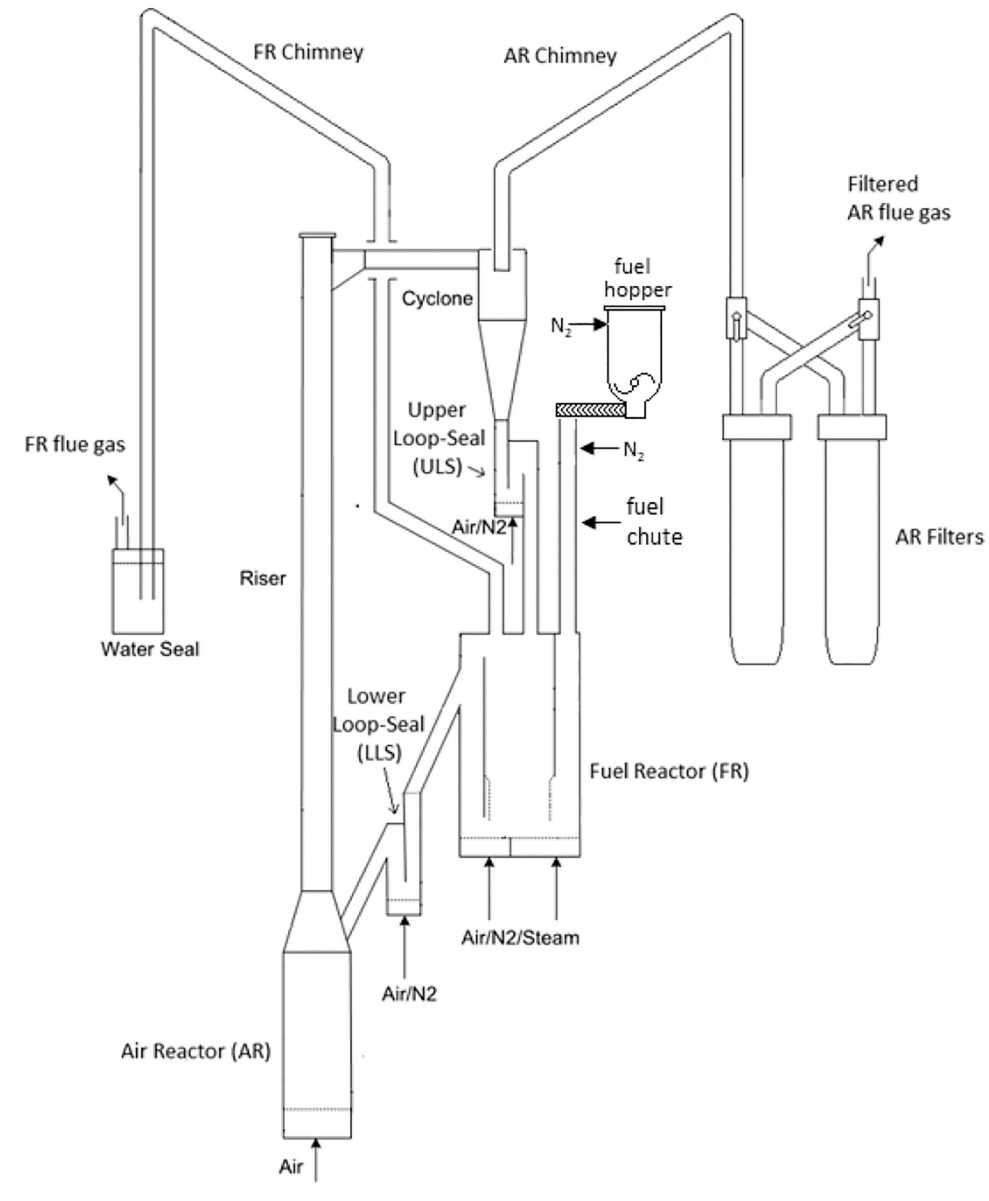
Simplified schematic of the 10 kW_th_ solid fuel CLC pilot. This figure was reprinted with permission from ref (31), Copyright 2021 Elsevier.

The 10 kW_th_ CLC pilot reactor system consists of a bubbling bed FR and a fast-fluidized bed AR. The FR is equipped with a volatile distributor, a perforated distributor mounted inside the FR that enhances contact between biomass volatiles and bed material. A diagram of the reactor system is shown in Figure 1. The reactor system is installed in an electrically heated oven, capable of heating the reactor up to 1000 °C. Setting the reactor oven temperature controls the operating temperature of the system. A more comprehensive overview of this CLC pilot can be found in previously published work.^31^

### 2.2 Oxygen Carrier

The 10 kW_th_ pilot was operated with LD slag oxygen carrier material. LD slag is an industrial waste byproduct of the Linz–Donawitz process, also known as the basic oxygen furnace process, where iron is processed into low-carbon steel. The LD slag material from SSAB AB was supplied in granular form, crushed to a size of <400 μm. The received material was calcined at 500 °C for 2 h and at 950 °C for 8 h. It was subsequently dry-sieved to reject fine particles that are less than 125 μm in diameter. Composition of the LD slag oxygen carrier is shown in Table 1.

**Table 1:** LD Slag Elemental Composition (Excluding Oxygen)

element	wt %
Fe	17
Ti	0.78
Ca	32
Si	5.6
Mg	5.9
Mn	2.6
V	1.5
Al	0.76
Cr	0.33
Ni	0.002
K	0.037

The LD slag oxygen carrier was selected because it is an inexpensive oxygen carrier material that showed reasonable CLC and CLG performance in earlier lab-scale investigations^33^ and pilot campaigns^34^ but exhibited lower interaction with ash materials and lower ability to capture potassium when compared to ilmenite.^25^ The lower capacity for potassium absorption is important for investigating the partitioning gas-phase alkalis between the FR and AR in the current study. All previous pilot investigations of alkali release from fuels were conducted with OC materials that exhibited strong absorption of potassium,^30−32^ making it difficult to draw conclusions on how the two-reactor scheme and the different fuel conversion processes of CLC systems affect the gas-phase release of alkalis from biofuels.

### 2.3 Fuels

Three biomass fuels were used in the experiments: BP, PFR, and SP. These fuels were selected because they cover a wide range of alkali content and have been used in previous investigations,^30−32^ thus providing a comparison basis. Table 2 summarizes the fuels used, their manufacturing and preparation processes, and the approximate fuel particle size. Fuel composition is summarized in Table 3.

**Table 2:** Manufacturing, Preparation, and Average Size of Biomass Fuels Used in the Campaign

fuel	supplier	manufacturing process	additional fuel preparation	approximate fuel particle size (as fed to the FR)
black pellets (BP)	Arbaflame AS, Norway	stem wood is steam-exploded, and resulting pulp is pelletized	pellets are crushed to a size of 0.7–2.8 mm	approximately 0.7–2.8 mm
pine forest residue (PFR)	National Renewable Energy Centre (CENER), Spain	pelletization of pine wood chips	pellets are crushed to a size of 0.7–3.0 mm	approximately 0.7–3.0 mm
straw pellets (SP)	Stohfelder, Austria	pelletization of wheat straw	wheat straw pellets are crushed to a size of 0.7–3.5 mm	approximately 0.7–3.5 mm

**Table 3:** Fuel Composition and Properties

parameter	unit	black pellets (BP)	pine forest residue (PFR)	straw pellets (SP)
moisture	wt %, as received	6.90	9.20	8.8
ash	wt %, as received	0.30	1.82	7.9
volatiles	wt %, as received	74.2	80.0	67.5
fixed carbon	wt %, as received	18.7	9.0	15.8
H	wt %, as received	5.6	5.69	6.1
C	wt %, as received	49.8	46.9	42.0
N	wt %, as received	0.09	0.347	0.70
O	wt %, as received	37.4	36.0	43.0
K	mg/kg of fuel, as received	460	2080	11000
Na	mg/kg of fuel, as received	<53	27	260
Cl	mg/kg of fuel, as received	<100	<100	1700
S	mg/kg of fuel, as received	<120	210	1080
Si	mg/kg of fuel, as received	<530	n/a	19000
Ca	mg/kg of fuel, as received	820	n/a	7700
LHV	MJ/kg, as received	18.6	18.0	15.1
K/Cl	atomic ratio	4.2	18.9	5.9
K/(Cl + 0.5S)	atomic ratio	2.5	8.8	4.4

### 2.4 Reactor Operation

The CLC pilot was operated in three different modes in this experimental campaign. Reactor operation was established by first starting fluidization of the AR and FR with air and warming the reactor system up to the operating temperature. Once sufficient solid circulation was established and the operating temperature was reached, FR fluidization was switched to nitrogen and then transitioned to steam. Once steam fluidization of the FR was established, fuel addition to the FR was initiated to start CLC tests. CLC operation was adjusted to maximize FR flue gas CO_2_ concentrations and minimize concentrations of CO, CH_4_, and H_2_. This was performed primarily by adjusting the AR air flow rate, which controls OC circulation in the system. CLC tests were conducted at reactor temperature set points of 870, 920, and 970 °C for BP and PFR fuels and at 870 and 970 °C for the SP fuel.

CLG tests were carried out in a fashion similar to CLC operation but with a much lower AR flow rate and, thus, lower OC circulation. CLG operation was signified by a lower FR CO_2_ concentration but higher FR CO and H_2_ concentrations. CLG operation was controlled by adjusting the fuel feed rate and the AR air flow rate, targeting a 1:1:1 ratio of CO/H_2_/CO_2_ in the FR flue gas. CLG tests were conducted at reactor temperature set points of 870, 920, and 970 °C for BP and PFR fuels and at 870 and 970 °C for the SP fuel.

OCAC tests were conducted by switching FR fluidization from steam to air while feeding fuel into the FR. Solid circulation between the AR and FR was maintained during OCAC operation. OCAC tests were carried out at the reactor temperature set point of 920 °C for BP fuel and at 970 °C for the PFR and SP fuels. Further toward CLC, CLG, and OCAC operations, several shorter tests were conducted to test the effect of the FR steam concentration on CLC and OCAC operations.

### 2.5 Flue Gas Alkali Measurement System

**Figure 2:**
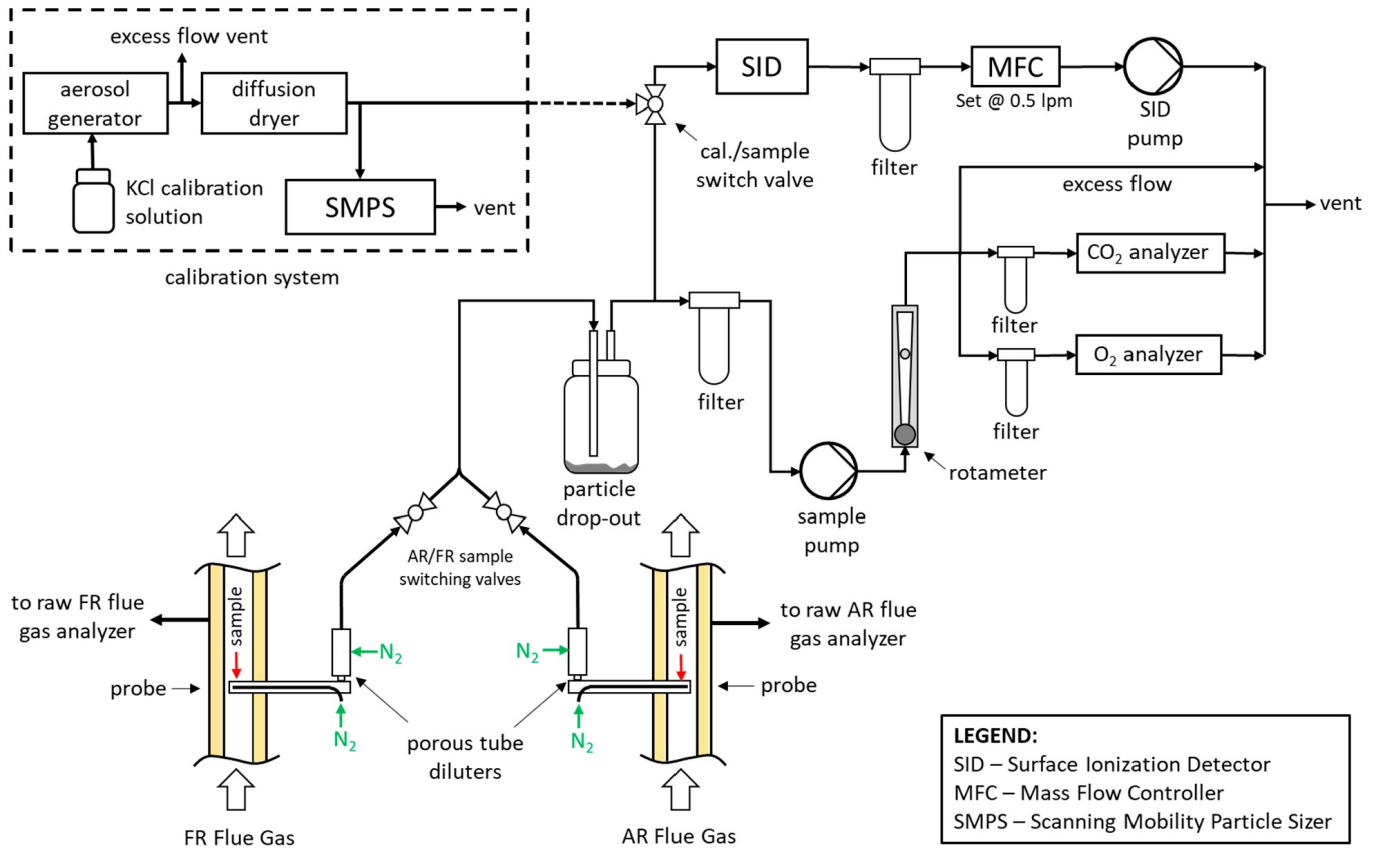
Flue gas alkali measurement system schematic.

The 10 kW_th_ CLC pilot reaction was equipped with a flue gas alkali emission measurement system. The schematic of this system is shown in Figure 2.

The setup shown in Figure 2 is capable of sampling from the FR or AR flue gas lines. Only one reactor can be sampled at one time, with switching between reactors facilitated by sample switching valves. Raw flue gas is sampled at the process temperature (870–970 °C) and is then diluted with nitrogen in two sequential stages before being delivered to the surface ionization detector (SID) and the trace CO_2_ and O_2_ analyzers. The first stage of dilution occurs inside the sampling probe that is installed right into the FR and AR flue gas lines. The probe is oriented such that the sample suction opposes the flow of the flue gas. This limits the ingress of oxygen carrier and fly ash particles, which are present in the flue gas. At the process temperatures of 870–970 °C, the flue gas alkali species of concern are present in the gas phase, negating the need for isokinetic sampling. The second stage of sample dilution occurs in a porous tube diluter that is installed immediately after the dilution probe tube exits the insulation of the FR of AR flue gas pipes. Dilution of the sample is carried out to cool the sample gas in a controlled manner, such that gaseous alkali aerosols nucleate into solid alkali aerosol particles. Controlled nucleation of solid alkalis is known to minimize sampling condensation loses that typically occur if the hot sample is allowed to be cooled by the sample line walls. Furthermore, because the FR flue gas can contain up to 80 vol % steam, dilution is required for preventing water from condensing in the sample lines. Lastly, dilution is required such that the diluted sample alkali concentration is within the calibration range of the SID instrument.

Sample suction in the system is created by a sample pump that is controlled by a rotameter with an integrated needle valve. After the sample passes through the probe and the porous tube diluter, the flow enters a drop-out vessel. The drop-out vessel is necessary to allow for fine LD slag particles carried with the FR flue gas to settle out. Earlier attempts of operating the CLC pilot with the LD slag oxygen carrier while sampling the flue gases for alkali emissions led to plugging up of the alkali sampling system within about 5 min of sampling. In these earlier tests, the suction of the sampling system was created with a venturi-type diluter, which forces the sample to flow through a small venturi orifice. This orifice could not tolerate the heavy LD slag particle loading of the sample flow. To resolve the plugging issue, the sampling system was modified to the configuration shown in Figure 2. The venturi diluter was replaced with a sample suction pump equipped with the particle drop-out vessel and a particle filter at the inlet of the sample pump. The drop-out vessel was designed to precipitate the major fraction of the fine LD slag particles, while the filter upstream of the sample pump took out the rest of the LD slag fines to protect the downstream pump and rotameter. Additional filters were added upstream of the trace CO_2_ and O_2_ analyzers as an extra precaution.

The trace CO_2_ (LICOR LI-850) and O_2_ (Alpha Omega Instruments 3000-Y230BTP) analyzers are used to track the overall dilution ratio of the sample. The trace CO_2_ analyzer has a measurement range of 0–20 000 ppm of CO_2_ and an accuracy of ±1.5% of the reading. The trace O_2_ analyzer is an auto-ranging three-range instrument (ranges of 0–100, 0–1000, and 0–10 000 ppm of O_2_) with an accuracy of ±1.0% of the selected range. For FR sampling, CO_2_ is used as a tracer gas. The FR sample dilution ratio is calculated by dividing the FR CO_2_ concentration by the CO_2_ concentration of the diluted sample. For AR sampling, O_2_ is used as a tracer gas. The AR dilution ratio is calculated by dividing the AR O_2_ concentration by the diluted sample O_2_ concentration. The dilution ratios are used in the data processing stage to recalculate the SID alkali measurements back to an undiluted raw flue gas basis.

To measure the alkali concentration, the SID draws a small unfiltered stream of the sample flow exiting the drop-out vessel. This stream is kept unfiltered to prevent loss of alkali aerosol before it reaches the SID. It should be noted that the solid alkali aerosol that forms in the dilution step is known to be in the sub-micrometer size range and would not be rejected in the particle drop-out vessel. In the SID, the alkali concentration of the diluted sample is measured using the principle of surface ionization. The method is highly sensitive and selective toward alkali compounds and has been successfully used for alkali measurement in conventional combustion,^16^ gasification,^16^ and previous CLC campaigns.^30−32^ A comprehensive overview of the working principles of the SID is described in several publications.^36−38^ The stream exiting the SID is filtered to protect the mass flow controller (MFC) and the SID suction pump.

Prior to use, the SID is calibrated with a KCl aerosol created with a TSI 3073 aerosol generator. The aerosol generator uses a 0.05 M KCl solution to create a flow of aqueous KCl aerosol in air. The aqueous aerosol flow is then passed through a diffusion dryer, where moisture is removed from the aqueous aerosol, precipitating solid KCl aerosol particles (10–600 nm range) in the air flow. The dried aerosol flow is split between the SID and a scanning mobility particle sizer (SMPS) system. The SMPS system (SMPS of TSI 3082 and CPC 3750) measures the mass concentration of the aerosol, while the SID reports a nanoamplifier signal that that is proportional to the alkali mass concentration. Several different aerosols of varying KCl concentration are generated by changing the pressure setting of the aerosol generator. A calibration curve for this SID instrument is generated by plotting the aerosol mass concentration that is reported by the SMPS versus the nanoamplifier signal reported by the SID.

In the present campaign, calibration of the SID was performed before the start and immediately after the end of reactor operation for each campaign day. The random error of the SID instrument was measured to be ±1.9% in a previous study.^39^ A systematic error of ±4% that is associated with the maximum instrument drift that occurs within a day of operation was estimated from comparison of the pre- and post-operation calibrations. From the combination of these two errors, the overall SID measurement uncertainty for days 4–6 of operation was estimated to be ±6%. Calibration issues were encountered with the SMPS system on days 1–3 of operation. These issues were mitigated by applying the average SID calibration results from days 4–6 to days 1–3. As a result of this substitution, an additional uncertainty of ±12%, corresponding to the maximum calibration deviation for 3 days of operation, was added to the base uncertainty of ±6%. As such, the SID measurement uncertainty for days 1–3 of operation was estimated to be ±18%. In interpretation of the experimental results, it is important to note that the aforementioned uncertainties are SID instrument uncertainties and do not account for alkali loses that occur in the sampling system. The exact impact of losses when sampling high-temperature combustion aerosols is difficult to determine and take into account^40^ but were shown to be low in a recent campaign using the sampling system implemented in the current study.^39^ As such, no corrections were implemented to account for sampling losses.

### 2.6 Flue Gas Analysis

In addition to the alkali emission measurement system, gas analysis of the AR and FR was critical for alkali measurement. Gas composition for the AR and FR flue gas was determined by multicomponent gas analyzers. Sampled flue gases were conditioned to remove solids and condense out water prior to being introduced into the analyzers. The gas analyzers, gas-conditioning systems, gases measured, and measurement uncertainties are summarized in Table 4.

**Table 4:** Flue Gas Analysis Key Equipment and Measurement Characteristics^a^

analysis	gas conditioning system	gas analyzer	measured component	measurement principle	measurement range (%)	measurement uncertainty (%)
FR flue gas composition	M&C Products SS-5	Rosemount NGA-2000 MLT4	CO	NDIR	0–100	<2.0
			CO_2_			
			CH_4_			
			H_2_	TC	0–20	<0.4
			O_2_	paramagnetic	0–100	<2.0
AR flue gas composition	M&C Products SS-5	SICK MAIHAK SIDOR	CO	NDIR	0–20	<2.0
			CO_2_	NDIR	0–5	<0.5
			O_2_	paramagnetic	0–25	<0.2

a Abbreviations: NDIR, non-dispersive infrared; TC, thermal conductivity.

## 3 Data Processing and Calculations

Reactor operational data and data reported by the alkali measurement system were recorded with a sampling rate of 1 Hz. The following subsections provide details on how the results and relevant process parameters were calculated.

### 3.1 Alkali Emissions

Alkali emissions on concentration basis were calculated by multiplying the signal reported by the SID, by the SID calibration constant (K_SID cal._) and the sampling dilution ratio (DR).
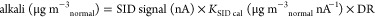
1Dilution ratios were calculated from raw flue gas tracer gas mole fractions (*X*_CO_2_,FR(dry)_ and *X*_O_2_,AR(dry)_) and the diluted sample tracer gas mole fractions (*X*_CO_2_,sample(dry)_ and *X*_O_2_,sample(dry)_).
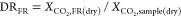
2
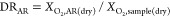
3Alkali emission concentrations were only calculated if the diluted sample concentrations of the tracer gases (CO_2_ and O_2_) were above 500 ppm. Tracer gas concentrations below 500 ppm were deemed unreliable because the CO_2_ and O_2_ trace analyzer error and reading instability become large at the lower end of the measurement range.

### 3.2 Carbon Gas Flow

The carbon gas parameter is a summation of flows of the main carbon-bearing gases detected in the FR flue gas. This parameter is calculated as a product of the sum of the CO, CO_2_, and CH_4_ mole fractions detected in the FR flue gas by the main process gas analyzers and the calculated value for the FR flue gas flow (*F*_FR(dry)_) on a dry basis.
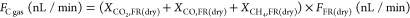
4The main process analyzers report dry basis concentrations of CO, CO_2_, CH_4_, H_2_, and O_2_ in the FR flue gas. It is assumed that the remainder of the dry flue gas fraction in the FR is made up of N_2_ that is introduced into the reactor loop seals as the fuel bin sweep gas and as sweep gas to several FR pressure taps. This assumption has been previously verified in earlier campaigns with direct measurement of the N_2_ concentration by a gas chromatograph. Because the flow rate of N_2_ flowing into the FR, *F*_N_2_,FR(dry)_, can be estimated as a summation of ^1^/_2_ of the N_2_ flows to loop seals and the measured N_2_ sweep gas flows, the FR flue gas flow on a dry basis can be calculated as follows:
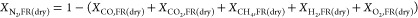
5
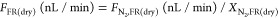
6

### 3.3 Oxygen Demand

The oxygen demand is the ratio of oxygen required to fully oxidize the flue gas of the fuel reactor to the stoichiometric oxygen required for complete fuel oxidation. The oxygen demand is defined as
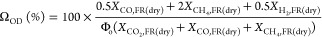
7Here, Φ_o_ is the O_2_/C molar ratio (mol of O_2_ required for combustion/kg of fuel)/(mol of C/kg of fuel).

### 3.4 Extent of Char Conversion

During fueled operation, fuel char conversion proceeds until the char particle is small and light enough to be elutriated via the FR chimney. The elutriated char was found in the FR chimney deposits. The extent of char conversion, η_carbon_, was estimated by dividing the carbon content of the FR chimney deposits that were collected during fueled operation by fixed carbon that was fed to the fuel reactor with the biomass fuel
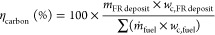
8where *m*_FR deposit_ is the mass of FR chimney deposits collected during fueled operation, *w*_c,FR deposit_ is the weight fraction of carbon in the FR chimney deposits, *m̈*_fuel_ is the mass flow rate of fuel into the system, and *w*_c,fuel_ is the mass fraction of fixed carbon in the fuel.

### 3.5 Retention of K by the OC Material

**Figure 3:**
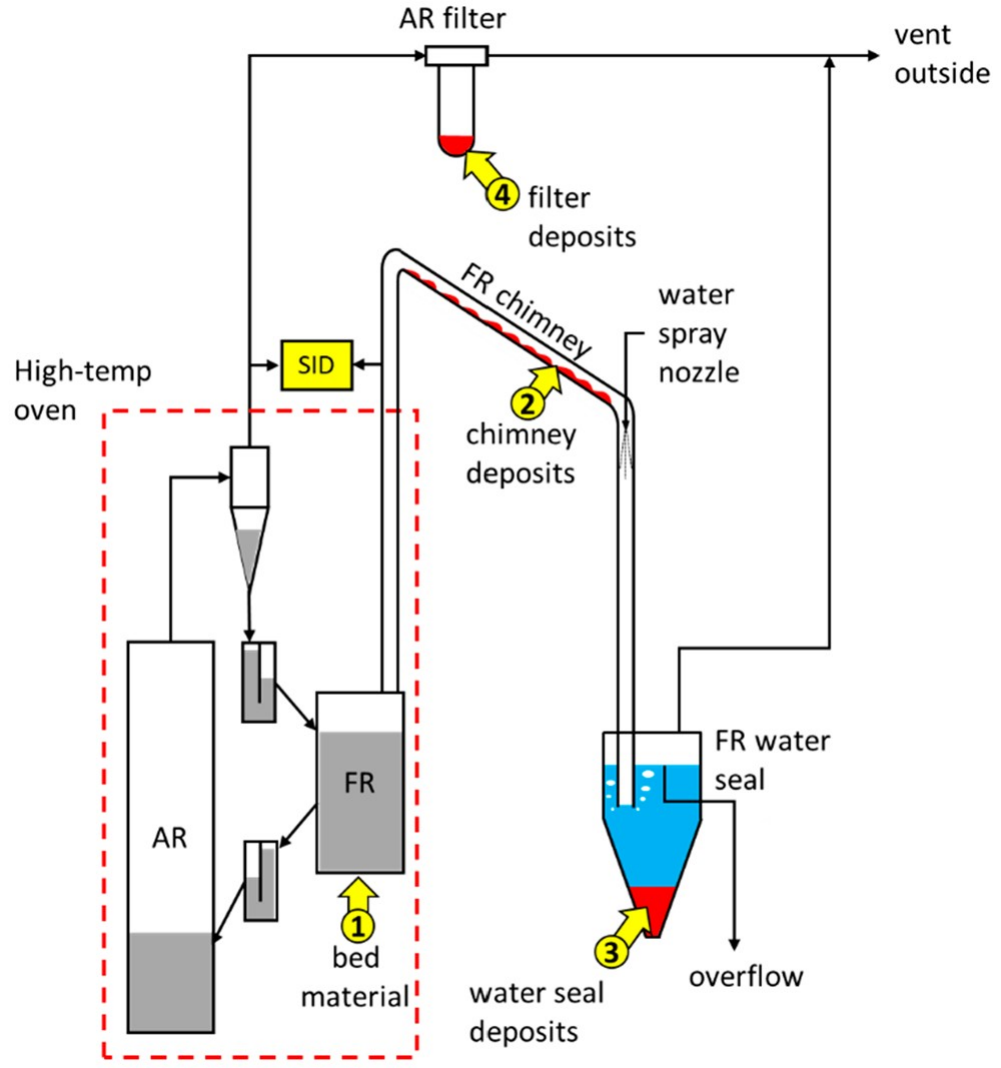
Solid sample locations of the 10 kW_th_ CLC pilot.

In continuous operation of the CLC pilot, the majority of the bed material circulates within the two-reactor system. During operation, some bed material is elutriated from the system. Elutriation occurs in the AR and FR. In the AR, solids that are not removed by the cyclone, end up captured by a downstream filter. The FR operates as a bubbling fluidized bed and is not equipped with a cyclone. Most of the material elutriated from the FR ends up depositing on the inner walls of the FR chimney. Some material that does not settle on the chimney walls ends up in the FR water seal. In the water seal, larger particles settle out into deposits, while smaller particles can either dissolve, if water-soluble, or remain suspended in the water until leaving the water seal via the water seal overflow. The possible locations for accumulation of solid particles in the reactor system are depicted in Figure 3. To understand how potassium is distributed in solid form, four types of solid samples were collected during reactor operation. The sampling locations are shown in Figure 3. The types of solid samples are (1) FR bed material samples, taken directly from the FR bed during operation; (2) FR chimney deposit samples, taken during periodic cleaning of chimney deposits; (3) FR water seal deposit samples, taken at the end of each day of operation; and (4) AR filter samples, taken during periodic AR filter changeover.

Type 1, 2, and 4 solid samples were analyzed by Eurofins AB for K content and in several cases for full elemental composition using inductively coupled plasma optical emission spectroscopy (ICP–OES). Solids that accumulate in the FR water seal (type 3) were not analyzed because this material is continuously washed with water that comes into the water seal as steam condensate from the FR flue gas as well as from a water spray nozzle installed in the downcomer of the FR chimney. It is expected that, in contact with water, a large portion of K in the solids would be leached out. In addition to elemental analysis, the particle size distribution (PSD) of sample types 1–3 was determined by sieving. Furthermore, sample types 1–4 were inspected with scanning electron microscopy (SEM) to visually inspect particle homogeneity as well as estimate the average size of the AR filter particles.

Elemental analysis of AR filter material showed an overall composition that is similar to the LD slag bed material but with a much higher K content. The size of the AR particles was determined to be in the range of 5–10 μm. This indicates that AR filter deposits consist of LD slag fines that form through attrition and are enriched with K either from condensation of K onto these fines within the AR chimney or by the presence of individual fuel ash particles within the AR filter material. Energy-dispersive X-ray analysis (EDX) mapping of the AR filter sample could not identify any clusters or areas of distinctly different composition, which would indicate separate ash particles. However, even fresh LD slag material is highly heterogeneous and contains the majority of the same elements as typically contained in biomass fuel ash. As a result of their small particle size and high K content, AR filter solids (sample type 4) were considered to be AR fly ash for the purpose of the K material balance.

For sample types 1–3, the elemental composition, PSD, and SEM analyses indicated that these samples essentially consist of the ash-free LD slag oxygen carrier with a slightly increased K content. Considering that the OC material is distributed into these three fractions, the amount of K retained by the OC over the period of fueled operation can be estimated as the difference in the K content of the OC after fueled operation and the K content of the OC before fueled operation.
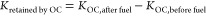
9In eq 9, the *K*_OC,after fuel_ term is a summation of K that is contained in various OC fractions after fueled operation. This term is defined as
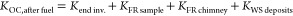
10where *K*_end inv._ is the mass of K in the reactor inventory at the end of the campaign day. Reactor inventory is tracked by accounting for all solid material additions and withdrawals from the system. The K content is taken from analysis of FR solid samples. *K*_FR sample_ is the mass of K removed from the reactor when taking FR bed samples. The FR sample mass is tracked, and the K content is taken from FR sample elemental analyses. *K*_FR chimney_ is the mass of K removed from the reactor and trapped in the FR chimney deposits. The FR chimney mass is recorded during periodic chimney deposit removal. The K concentration is determined from analysis of chimney deposits. *K*_WS deposits_ is the mass of K removed from the reactor and trapped in the FR water seal deposits. Water seal deposit mass was estimated at 1 kg/day of operation. The K concentration was assumed to be the same as in the FR chimney deposits.

In eq 10, the *K*_OC,before fuel_ term is a summation of the K content that is introduced into the system with the addition of bed material. This term is defined as
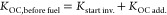
11where *K*_start inv._ is the mass of K in the reactor inventory at the start of the campaign day. The reactor inventory is tracked by accounting for all solid material additions and withdrawals from the system. The K content is taken from elemental analysis of FR solid samples. *K*_OC add._ is the mass of K added to the system with periodic additions of OC to the reactor. OC additions to the reactor are carefully tracked. The K content of the added material is determined from material elemental analyses.

Considering the above-defined parameters, the retention of K by the OC, relative to the K input with the biomass fuel, can be estimated as follows:
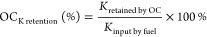
12

## 4 Results and Discussion

Alkali emission measurement with the SID-based system proved to be quite challenging to carry out. Process fluctuations, such as pressure fluctuations or fluctuations in the fuel feeding rate, made stable flue gas sampling and conditioning difficult to maintain. The error in alkali measurements was minimized in the data processing stage by omitting measurement data generated during operational upsets, which manifested in unstable sample dilution. Average alkali emissions for the tests conducted in this investigation are summarized in Table 6. Alkali emissions measured by the SID are reported on several different bases. Alkali emissions are first reported on a fuel-specific basis in mg of KCl_eq_/kg of fuel and on a concentration basis in mg of KCl_eq_/m^3^_n_. Both report alkali in KCl_eq_ terms because the SID response is calibrated with a KCl aerosol. These bases are useful for relative comparison of alkali emissions across different test conditions. However, results presented in KCl_eq_ terms are not well-suited to indicate actual amounts of alkalis present in the flue gases. Recent tests have shown that the SID response to different potassium salts can vary. Thus, a given SID response may arise from different amounts of different alkali species. Potassium (K) is the dominant alkali element in biomass conversion because it typically exists in large excess to sodium (Na) in most biomass fuels. Henceforth, the discussion focuses on K as the key alkali element of concern, and the terms “alkali” and “potassium/K” are used interchangeably. SID response to predominant K species that occur in combustion systems was explored in a recent study by Gall et al.^39^ and was further validated during pre-campaign calibration of the SID instrument. The approximate signal response factors are summarized in Table 5. The response factor is a relative measure of the response signal of the SID to the amount of K contained in an alkali species. The units of the SID response factor are (nA/μg of K)_salt_/(nA/μg of K)_KCl_. The response factor is normalized for the response of the SID to KCl, because KCl is the calibration salt used for SID calibration.

**Table 5:** SID Response Factors for Different Alkali Species

predominant K species	SID response factor [(nA/μg of K)_salt_/(nA/μg of K)_KCl_]
KCl	1
KOH/K_2_CO_3_	0.3
K_2_SO_4_	0.13

If the approximate speciation of gas-phase K is known, the factors from Table 5 can be used to estimate a range of K that is detected in the flue gases by the SID. In current experiments, the amount of K associated as K_2_SO_4_ in the flue gas is expected to be insignificant, because K_2_SO_4_ is unlikely to form in reducing conditions.^41^ The majority of the gas-phase alkalis are expected to occur as KCl, as far as Cl availability allows, with the remainder of K likely to exist as KOH(g) in the flue gas and as K_2_CO_3_ as the sample flow is cooled in the presence of CO_2_ within the SID sampling system. Thus, the amount of gas-phase K contained in the flue gas can be better estimated as a range using the response factors for KCl and KOH/K_2_CO_3_. This approach is used in Table 6 to estimate the approximate percentage of gas-phase alkalis detected in the flue gas of each reactor. The lower range value corresponds to the mass of K, assuming all alkalis in the flue gases are present in the form of KCl. The upper range value corresponds to the mass of K, assuming that all alkalis in the flue gas are present in the form of KOH/K_2_CO_3_.

**Table 6:** Average Alkali Emission Results and Relevant Test Parameters

day	fuel	test mode	temperature set point (°C)	duration (hh:mm)	FR temperature (°C)	FR alkali (mg of KCl_eq_/kg of fuel)	FR alkali (mg of KCl_eq_/m^3^_n_)	FR alkali (% of fuel K)	AR temperature (°C)	AR alkali (mg of KCl_eq_/kg of fuel)	AR alkali (mg of KCl_eq_/m^3^_n_)	AR alkali (% of fuel K)
1	BP	CLC	870	02:21	873	8.6	3.0	1.0–3.3	843	14.0	1.8	1.6–5.3
			920	00:59	923	19.4	6.7	2.2–7.4	896	10.6	1.6	1.2–4.0
		OCAC	920	00:17	921	6.9	2.3	0.8–2.6	861	5.7	0.8	0.7–2.2
2	BP	CLC	970	02:05	973	43.6	10.6	5.0–16.5	969	16.6	1.3	1.9–6.3
		CLG	970	00:55	973	48.7	11.2	5.6–18.5	953	13.2	1.3	1.5–5.0
3	PFR	CLC	870	00:23	878	6.7	2.1	0.2–0.6	889	6.3	0.7	0.2–0.5
			920	00:11	927	19.0	5.4	0.5–1.6	930	6.4	0.7	0.2–0.5
			970	00:51	974	31.2	7.2	0.8–2.6	968	11.1	0.7	0.3–0.9
		CLG	870	01:10	874	8.2	2.5	0.2–0.7	863	4.1	0.5	0.1–0.3
			920	00:42	925	19.0	5.8	0.5–1.6	910	5.1	0.8	0.1–0.4
			970	00:43	974	18.5	5.6	0.5–1.6	961	6.2	0.9	0.2–0.5
		OCAC	970	00:26	973	4.6	1.0	0.1–0.4	963	8.4	0.6	0.2–0.7
4	BP	CLG	870	01:27	875	2.8	1.1	0.3–1.1	870	2.8	0.5	0.3–1.1
			920	01:18	923	9.6	3.4	1.1–3.6	910	3.6	0.7	0.4–1.4
			970	02:02	973	20.4	6.0	2.3–7.8	956	3.3	0.5	0.4–1.2
5	PFR	CLG	870	00:45	874	9.4	3.2	0.2–0.8	851	3.5	0.6	0.1–0.3
			920	00:52	923	15.6	5.3	0.4–1.3	892	2.8	0.4	0.1–0.2
			970	02:39	972	19.0	6.0	0.5–1.6	948	2.6	0.4	0.1–0.2
6	SP	CLC	870	01:33	877	33.5	9.3	0.2–0.5	863	24.4	2.0	0.1–0.4
			970	02:02	973	153.7	41.9	0.7–2.4	957	19.1	1.6	0.1–0.3
		CLG	970	01:48	972	156.7	45.4	0.7–2.5	919	18.1	2.2	0.1–0.3
		OCAC	970	00:25	969	32.0	9.9	0.2–0.5	938			

### 4.1 Fuel Reactor Alkali Emissions versus Reactor Temperature

**Figure 4:**
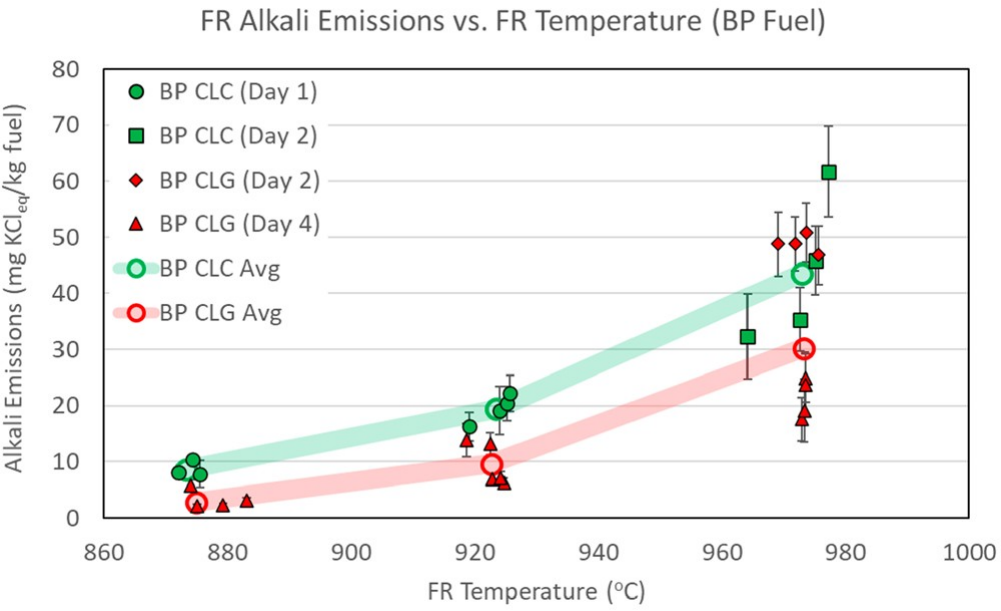
FR alkali emissions, with CLC and CLG operations with BP fuel.

**Figure 5:**
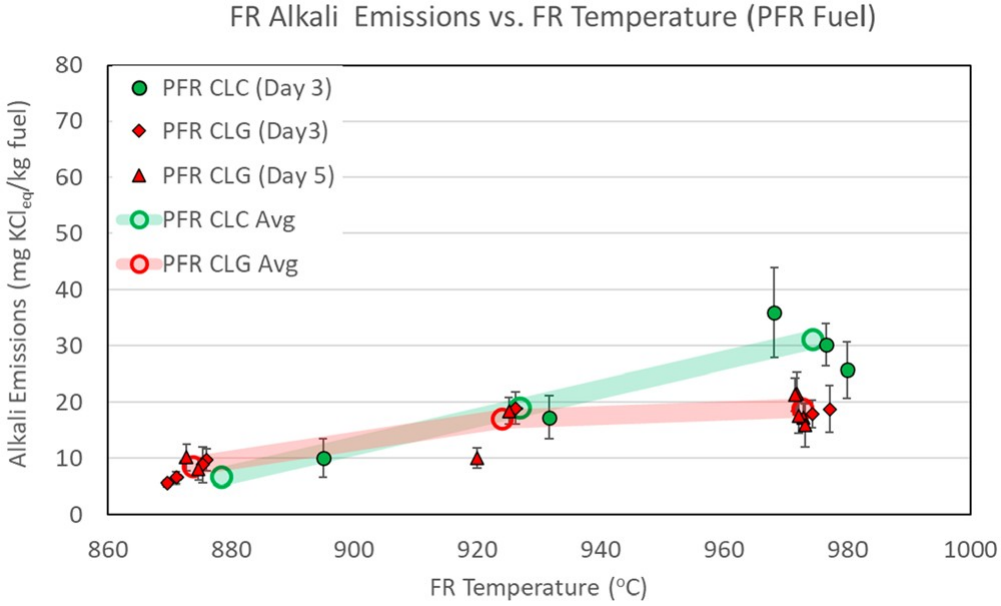
FR alkali emissions, with CLC and CLG operations with PFR fuel.

**Figure 6:**
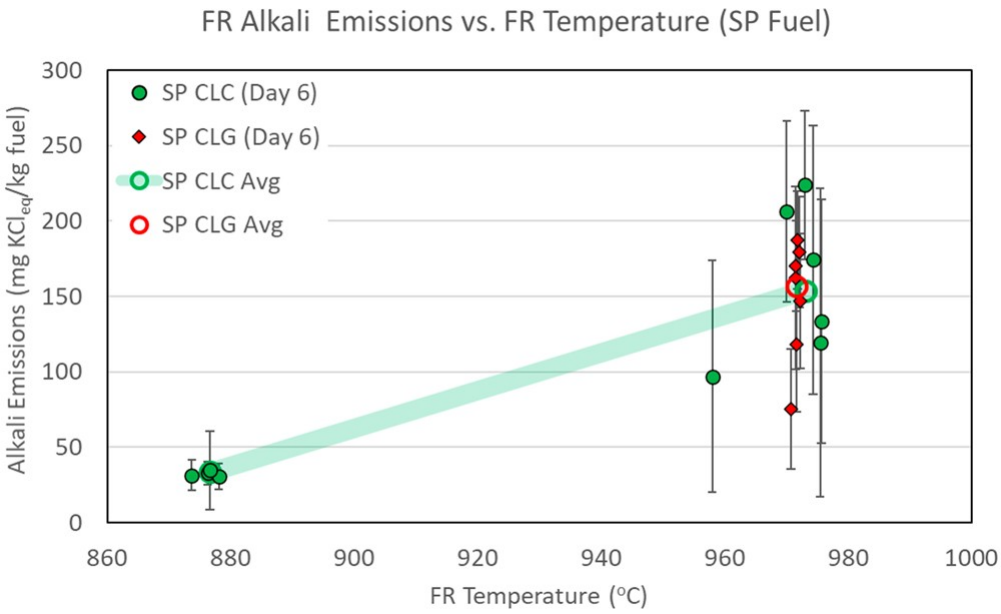
FR alkali emissions, with CLC and CLG operations with SP fuel.

Determining the temperature dependence of alkali emissions in CLC and CLG operations was the main focus of the campaign. Average FR alkali emissions measured for the different fuels and operating temperatures are presented in Table 6 and Figures 4–6. The figures include both CLC and CLG tests. Alkali emission numbers are normalized to the fuel feed rate and are expressed in units of mg of KCl_eq_/kg of fuel. Emission figures in Figures 4–6 are shown as average values for individual sampling periods. The data series are separated by type of operation (CLC shown in green and CLG shown in red) and by day of operation (each day shown in different marker shapes). The error bars reflect measurement data spread at one standard deviation of the measurements within each sampling period. The overall average emission figures for all sampling periods are also shown as open circles connected by semi-transparent lines to highlight the main data trends.

Figures 4–6 indicate that FR alkali emissions rise with the FR temperature in CLC and CLG operations. This trend is evident for all three biomass fuels and is in line with the initial hypothesis based on previous work and theory of alkali release. The literature on alkali release in conventional combustion, pyrolysis, and gasification indicates that <10% of K is released in the devolatilization step of fuel conversion, where organically associated K is released as KOH(g) or as part of the tar fraction.^19,21^ The majority of the gas-phase release of K occurs in the char conversion phase as the fuel char is heated to the reactor temperature. Dependent upon the fuel or specifically the form in which K is present in the char, the release is known to occur via several key pathways. In high-chlorine biomass, such as SP fuel in this study, a large proportion of K is known to occur as KCl in the char. As the fuel/char particle is heated beyond 700 °C, the vapor pressure of KCl becomes significant and rises sharply with the temperature. Evaporation of KCl is known to be the dominant mechanism for gas-phase release of alkalis in high-chlorine biomass.^19−21,42^ In low-chlorine biomass, such as BP and PFR fuel in this study, the majority of alkalis remaining in the char after fuel devolatilization are present in the form of carbonates, sulfates, and silicates, listed in decreasing order of importance.^19^ These forms can also hold a significant amount of K that is present in excess of KCl in high-chlorine biomass. Dependent upon conditions, potassium carbonate, sulfate, and silicate can decompose to release KOH(g) or K(g) in high-temperature environments. The onset and rate of decomposition of these compounds is highly temperature-dependent. In their modeling and experiments, Knudsen et al.^20^ estimated that the decomposition rate of these compounds increases roughly 10-fold for every 100 °C of temperature rise. Their experiments also showed that potassium sulfate and silicate decomposition become significant at temperatures above 1000 °C, while decomposition of K_2_CO_3_ becomes significant at temperatures exceeding 800 °C. Because the current system was operated in the range of 820–970 °C, K_2_CO_3_ decomposition is likely the main pathway for gas-phase alkali release during conversion of BP and PFR fuel char. Furthermore, second only to alkali release by KCl sublimation, K_2_CO_3_ decomposition is likely the next most dominant pathway for gas-phase alkali in conversion of SP fuel char. With both the KCl evaporation and K_2_CO_3_ decomposition mechanisms being highly temperature-dependent, a higher reactor temperature significantly increases the overall alkali release to the gas phase of the FR. Experimental results presented in Figures 4–6 clearly demonstrate and validate the influence of the reactor temperature on FR alkali emissions.

In comparison of the emissions between fuels, it is surprising to see that BP and PFR alkali emissions seem to be on similar levels for operating temperatures of 870 and 920 °C and BP alkali emissions are up to 30% higher at 970 °C. PFR fuel contains approximately 4 times more K than PB fuel, and a previous study conducted with these same fuels but with ilmenite oxygen carrier showed that PFR alkali emissions were approximately 20–40% higher than those for BP fuel.^31^ The tests in this previous study were conducted in the same pilot unit and similar conditions. The only significant difference between these two test campaigns was the different OC bed material. It is possible that the discrepancy in the relative alkali emissions of BP and PFR fuels can be related to the different alkali absorption mechanisms of ilmenite and LD slag oxygen carriers. Unfortunately, the exact reason for the discrepancy could not be established.

### 4.2 Air Reactor Alkali Emissions versus Reactor Temperature

**Figure 7:**
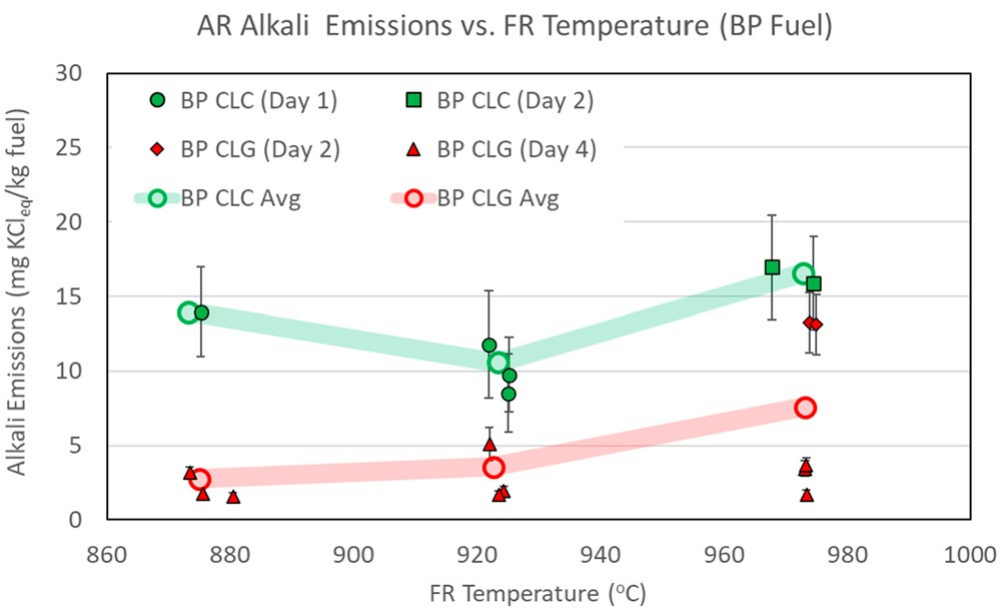
AR alkali emissions, with CLC and CLG operations with BP fuel.

**Figure 8:**
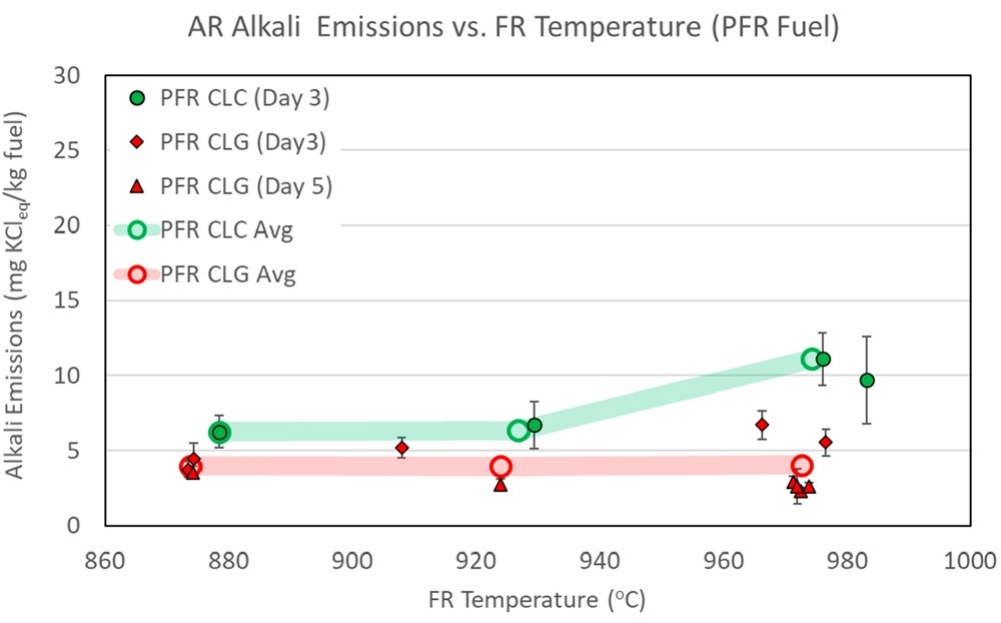
AR alkali emissions, with CLC and CLG operations with PFR fuel.

**Figure 9:**
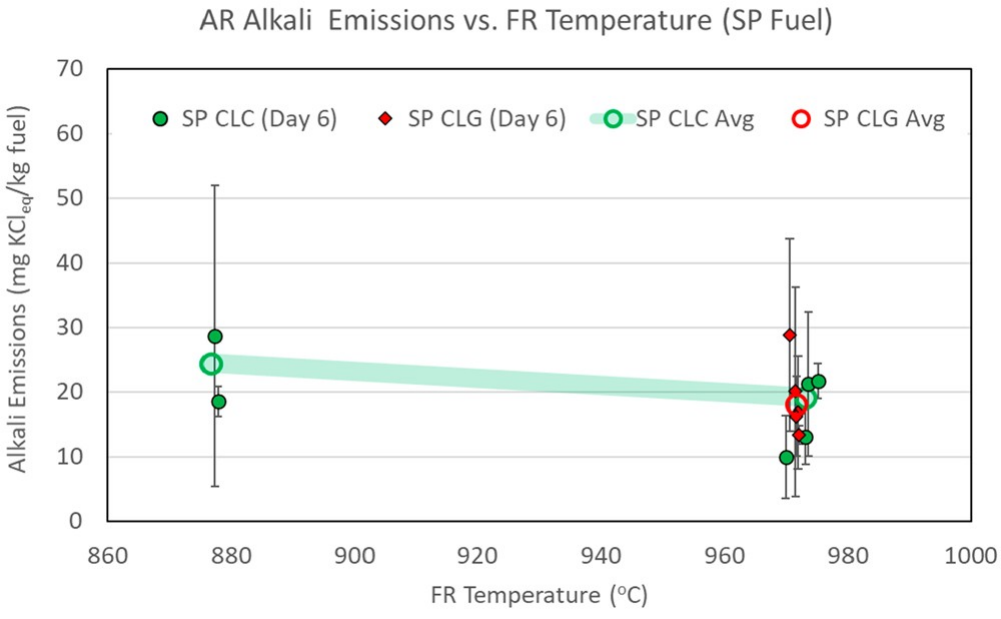
AR alkali emissions, with CLC and CLG operations with SP fuel.

AR alkali emissions measured in CLC and CLG operations are presented in Figures 7–9. The data in the figures are separated by type of operation (CLC versus CLG) and by day of operation. Each data point shows an average of emissions for individual sampling periods. The error bars reflect measurement data spread at one standard deviation of the measurements within each sampling period. The overall average emission figures for all sampling periods are also shown as open circles connected by semi-transparent lines to highlight main data trends.

Figures 7–9 show that AR alkali emissions remain approximately constant for CLC and CLG operations at the three FR temperature levels. With respect to comparison of AR alkali emissions to those of the FR, Table 6 shows that, at the lowest operating temperature set point of 870 °C, approximately similar amounts of alkalis are released in the AR as in the FR per kilogram of fuel fed into the pilot system. At the two higher temperature set points, the FR release exceeds the AR alkali release. In comparison of alkali release rates, however, it is more useful to look at the alkali emissions on the basis of the alkali concentration in the flue gases of the two reactors. Table 6 shows that AR flue gas alkali concentrations are consistently low and are significantly lower than FR flue gas alkali concentrations, even at the lowest operating temperature. As such, an approximately similar overall AR and FR alkali release at the lower operating temperature occurs as a result of a much higher flow rate of the AR. Looking across all test conditions with all three fuels, the AR alkali concentration does not corelate with the reactor temperature and does not change significantly between CLC and CLG operating modes for PFR and SP fuels.

In a previous investigation of alkali emissions in a dual interconnected circulating fluidized bed (D-CFB) CLC pilot, AR alkali emissions were found to be significant and, in several cases, higher than those in the FR. However, AR alkali emissions in the D-CFB study were determined to occur mostly as a result of carryover of unconverted char from the FR to the AR.^32^ In CLC systems, char carryover to the AR can be detected by monitoring the AR CO_2_ concentration, because char readily burns in the AR, releasing CO_2_. A review of AR CO_2_ concentrations in the present tests concluded that no significant carryover of char from the FR to the AR occurred during CLC or CLG operation. Thus, the AR alkali emissions in the current experiments must be caused by carryover of alkalis that are bound to the OC in the FR and re-release in the AR or are transported in the form of ash that originates in the FR and is carried over to the AR. One possibility is that decomposition of alkali salts to gaseous alkali species does not proceed to completion in the FR as a result of a residence time limitation imposed by the OC circulation. When carried over to the AR, these salts can continue to decompose to the gas phase of the AR. Another possibility is that certain condensed phase alkali species decompose to gaseous alkalis in the FR up to the equilibrium levels, thus completing the release, but when carried over, the AR undergoes further decomposition as a result of the slightly higher temperature of the AR or the oxidizing atmosphere of the AR. The exact pathway of such carryover could not be established in this investigation.

### 4.3 Fuel Reactor Alkali Release—CLC versus CLG Operation

**Figure 10:**
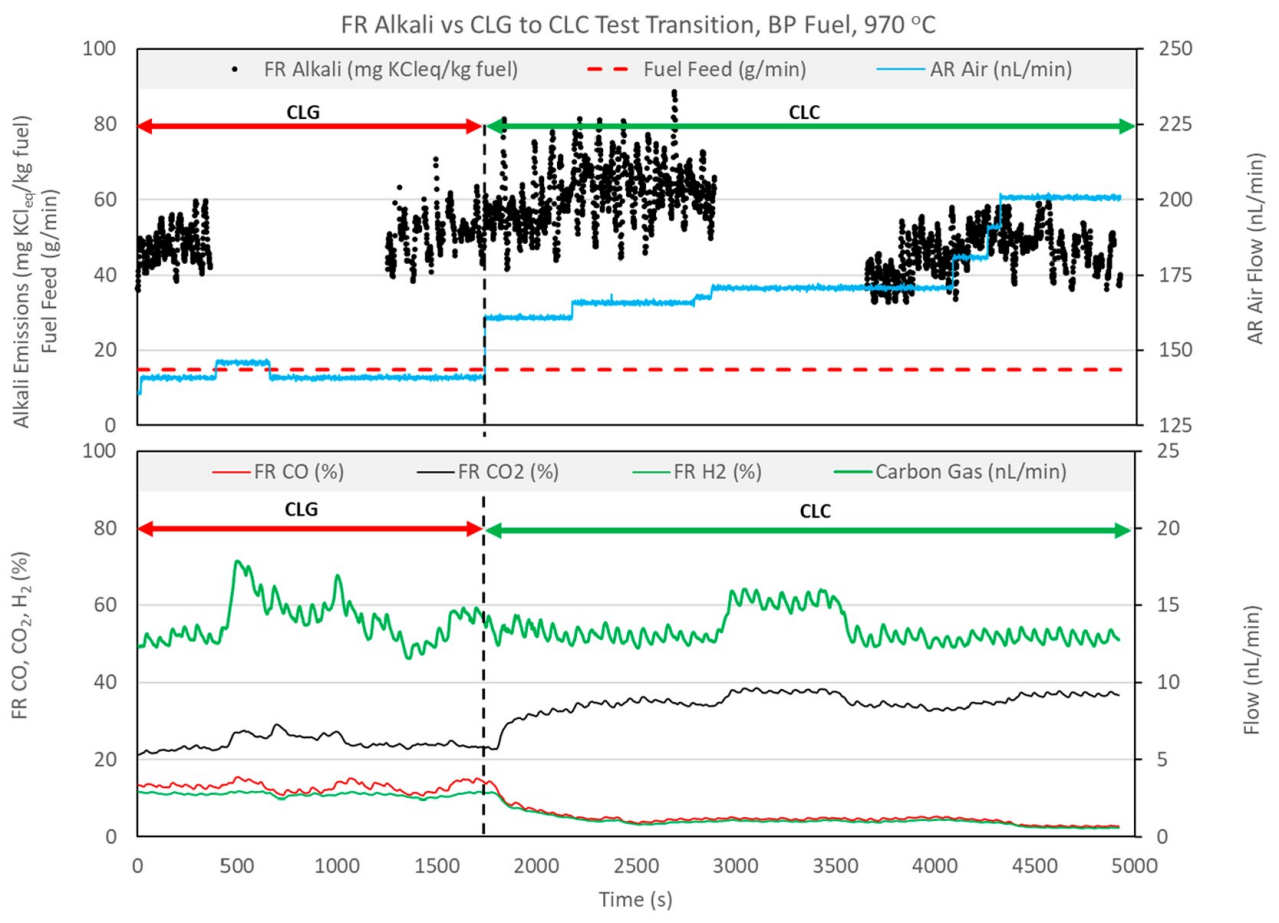
Effect of CLG to CLC transition on FR alkali emissions, with BP fuel at 970 °C.

**Figure 11:**
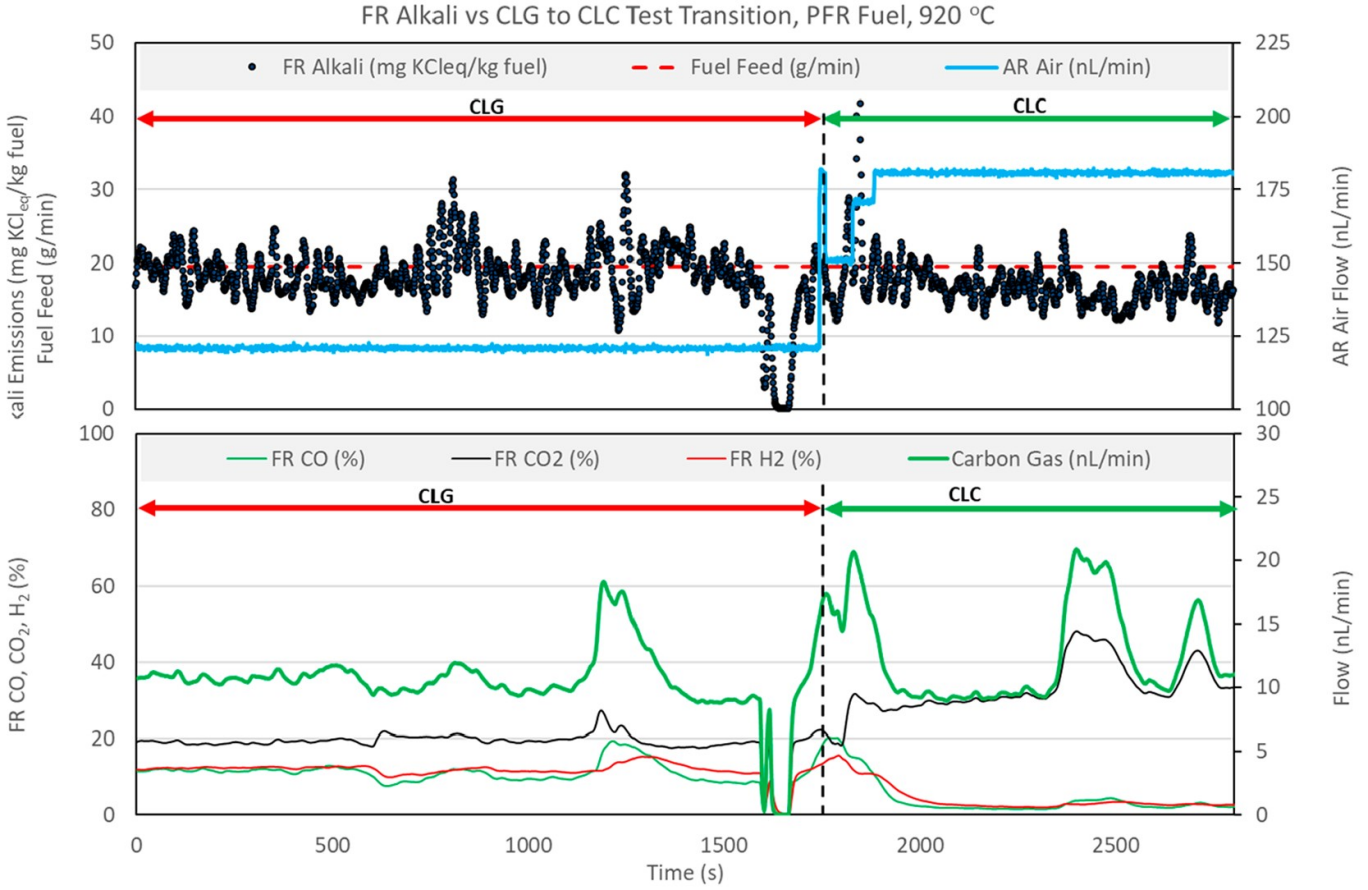
Effect of CLG to CLC transition on FR alkali emissions, with PFR fuel at 920 °C.

The effect of CLC versus CLG operating mode on FR alkali release is depicted in Figures 4–6. These figures show that FR alkali emissions in CLC and CLG modes were quite similar for PFR and SP fuels. Average CLG FR alkali emissions are slightly lower than average CLC FR emissions for BP fuel. However, it is important to note that CLC and CLG operations for BP fuel were conducted on different operation days, and some of the difference may be caused by the high measurement uncertainty of the SID on days 1 and 2 of operation. To better evaluate the effect of CLG versus CLC operation, it is most useful to look at CLC and CLG tests conducted within the same day of operation. Figures 10 and 11 show FR alkali emissions and relevant process parameters for operation segments where CLG operation transitions to CLC operation.

In Figures 10 and 11, transition from CLG to CLC operation is achieved by changing the air flow rate to the AR. The AR flow rate directly controls OC circulation and, thus, the oxygen delivery to the FR. This is evident from the decrease in CO and H_2_ concentrations and an increase of the CO_2_ concentration in the FR in response to the increased AR air flow rate. In operation with BP fuel (Figure 10), the initial transition from CLG to CLC operation seems to result in a slight rise in FR alkali emissions. However, FR alkali emissions later return to levels approximately equal to those in earlier CLG operation. In operation with PFR fuel (Figure 11) the transition from CLG to CLC is more drastic, with the AR air flow rate increased by 50% in a single step. Again, the transition to CLC is evident as CO and H_2_ concentrations fall and the CO_2_ concentration rises. The FR alkali emission levels appear to be unaffected by the change in operating mode. The sudden drop in alkali emissions that occurs at approximately 1600 min is caused by a temporary fuel feeding stop.

Literature on gas-phase alkali in thermal conversion of biomass attributes the vast majority of alkali release to the char conversion step. Thus, a difference in alkali release can be expected if CLC and CLG operations vary in terms of char conversion. The rate of char conversion in CLG operation should be slower than in CLC. Because H_2_ and CO concentrations in the FR product gas are much higher in CLG operation, char conversion is subject to H_2_ and CO inhibition.^43,44^ The extent of char conversion, however, is mostly controlled by char elutriation from the FR, because char carryover in the AR was confirmed to be insignificant. In both, CLC and CLG modes, char particles continue to convert and reduce in size and density up to a point where this char particle is small and light enough to be elutriated into the FR chimney. Elutriation of solids from the FR is known to occur in the current system because deposits of solid material (bed material, fly ash, and unconverted char) settle and build up on the inner walls of the FR chimney during operation. Figures 10 and 11 include a calculated carbon gas flow rate, expressed in nL/min. This parameter is calculated as the summation of the flows of CO_2_, CO, and CH_4_ measured in the exhaust of the FR. The value of carbon gas can be used for relative comparison of char conversion in the two modes of operation. In both processes, a large share of the carbon-bearing gases arises from the volatile content of the fuel, because fixed carbon makes up 37.6% of total carbon for BP fuel, 19.6% for PFR fuel, and 36.6% for SP fuel. Because the reactor temperature, fuel, and fuel feed rate are the same for each CLG-CLC test pair, the contribution to carbon-bearing gases from devolatilization of fuel should be similar in CLG and CLC cases. The residual portion of the carbon-bearing gases arises from char conversion. With a fixed contribution from devolatilization, difference in values for the carbon gas parameter should be a reasonable indication of the difference in char conversion. The average value of carbon gas flow in Figure 10 remains relatively constant across the CLG–CLC test pair, indicating that average char conversion is approximately similar in CLG and CLC operations. In Figure 11, the average value of carbon gas is also approximately equivalent in CLG and CLC operations. Three significant spikes are observed in Figure 11, each lasting from 2 to 3 min of operation. These spikes occur as a result of the short-lived increase in fuel feeding that is likely caused by a buildup and consequent instantaneous re-release of fuel from the fuel feed screw. This abrupt injection of fuel results in a significant increase in devolatilization products but also an increased amount of char being converted in the time interval of the disturbance. The alkali emission data shown in Figure 11 are normalized to 1 kg of fuel with the assumption of a constant fuel feeding rate. Thus, if alkali release is dependent upon char conversion, the alkali signal should follow the shape of the peaks of the carbon gas parameter. From the data shown in Figure 11, it is difficult to judge if this is the case.

To estimate the extent of char conversion more objectively, samples of FR chimney deposits were analyzed for carbon content. The mass of carbon found in the chimney deposits was used to estimate the overall extent of char conversion, as per the methodology outlined in section 3.4. The results are presented in Table 7.

**Table 7:** Char Conversion Estimates from FR Chimney Deposit Analysis

fuel	mode	temperature set point (°C)	char conversion (%)
BP	CLC	870	98
		920	98
	CLG	870	99
PFR	CLC	870	95
		920	97
		970	98
	CLG	970	97
SP	CLC	870	97
		970	96
	CLG	970	99
	OCAC	970	98

Although the accuracy of char conversion figures presented in Table 7 is subject to significant uncertainty, the calculated values suggest that overall char conversion was high and roughly equivalent for different operating modes. Given that the carbon gas parameter and more direct chimney deposit-based estimates indicate that char conversion was similar in CLC and CLG cases, we conclude that FR alkali emissions in CLC and CLG modes are similar due to similar levels of char conversion achieved in these two modes of operation.

### 4.4 Alkali Release in CLC versus OCAC

**Figure 12:**
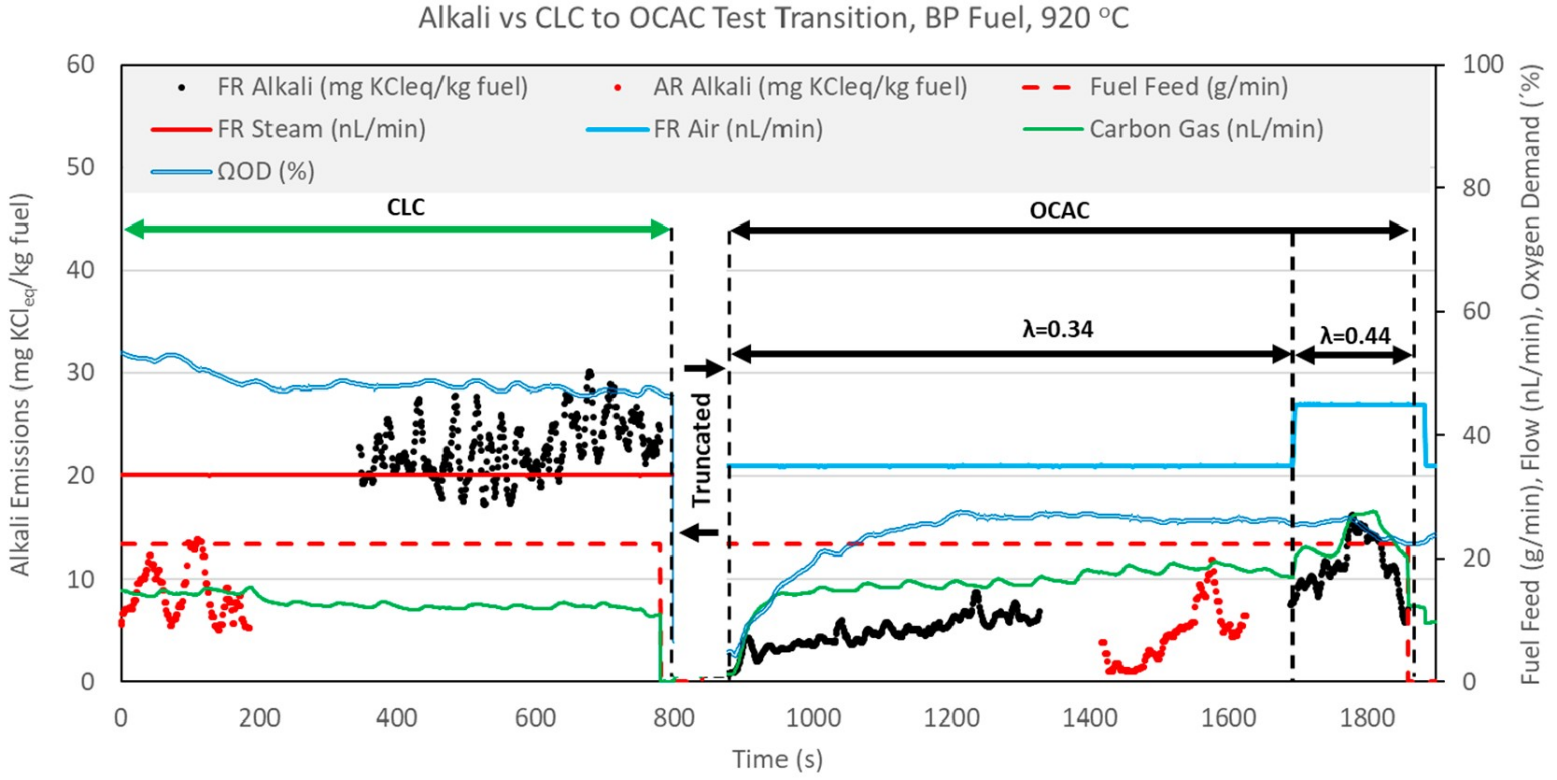
CLC versus OCAC alkali emissions for BP fuel operation at 920 °C.

**Figure 13:**
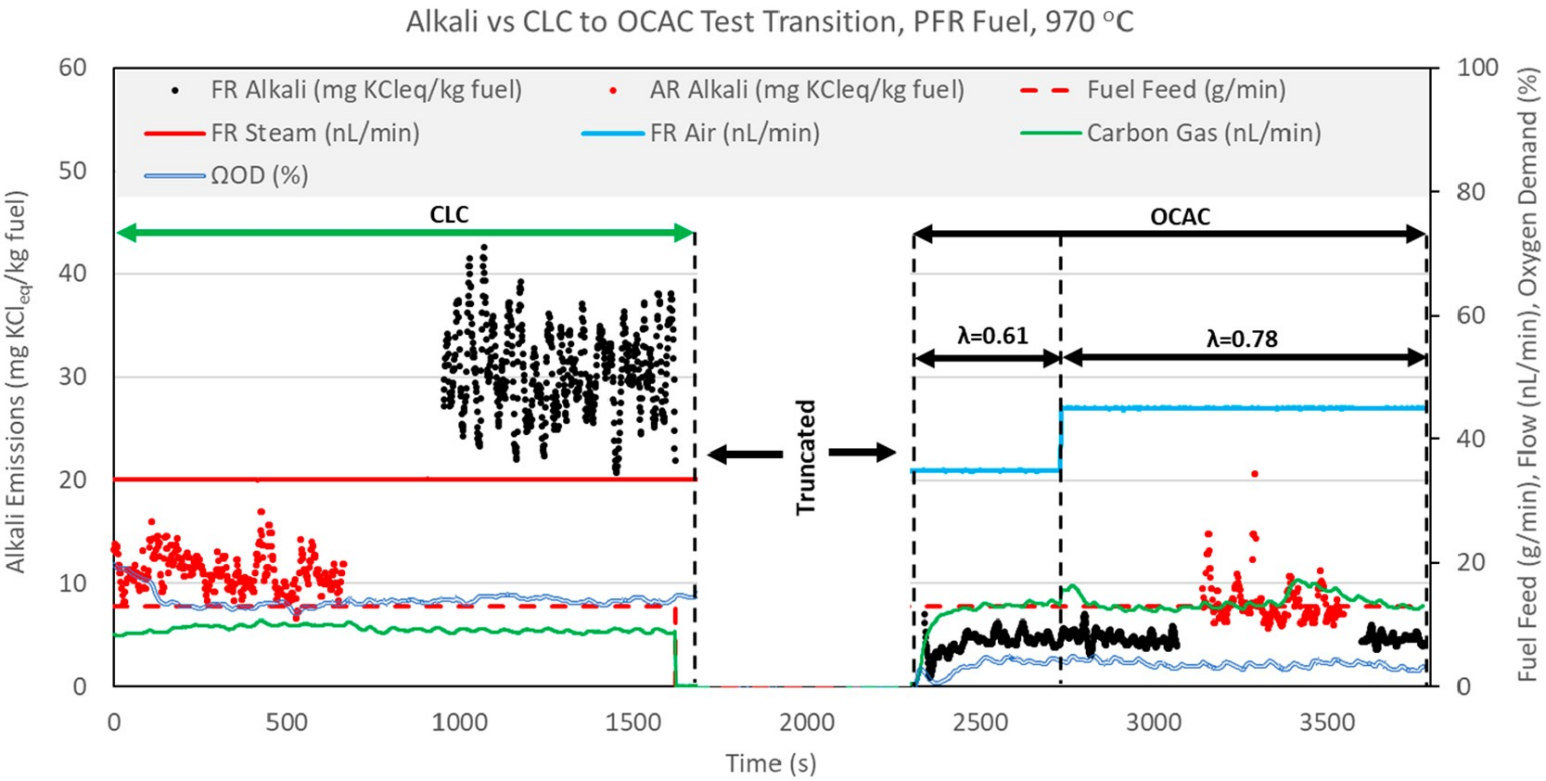
CLC versus OCAC alkali emissions for PFR fuel operation at 970 °C.

**Figure 14:**
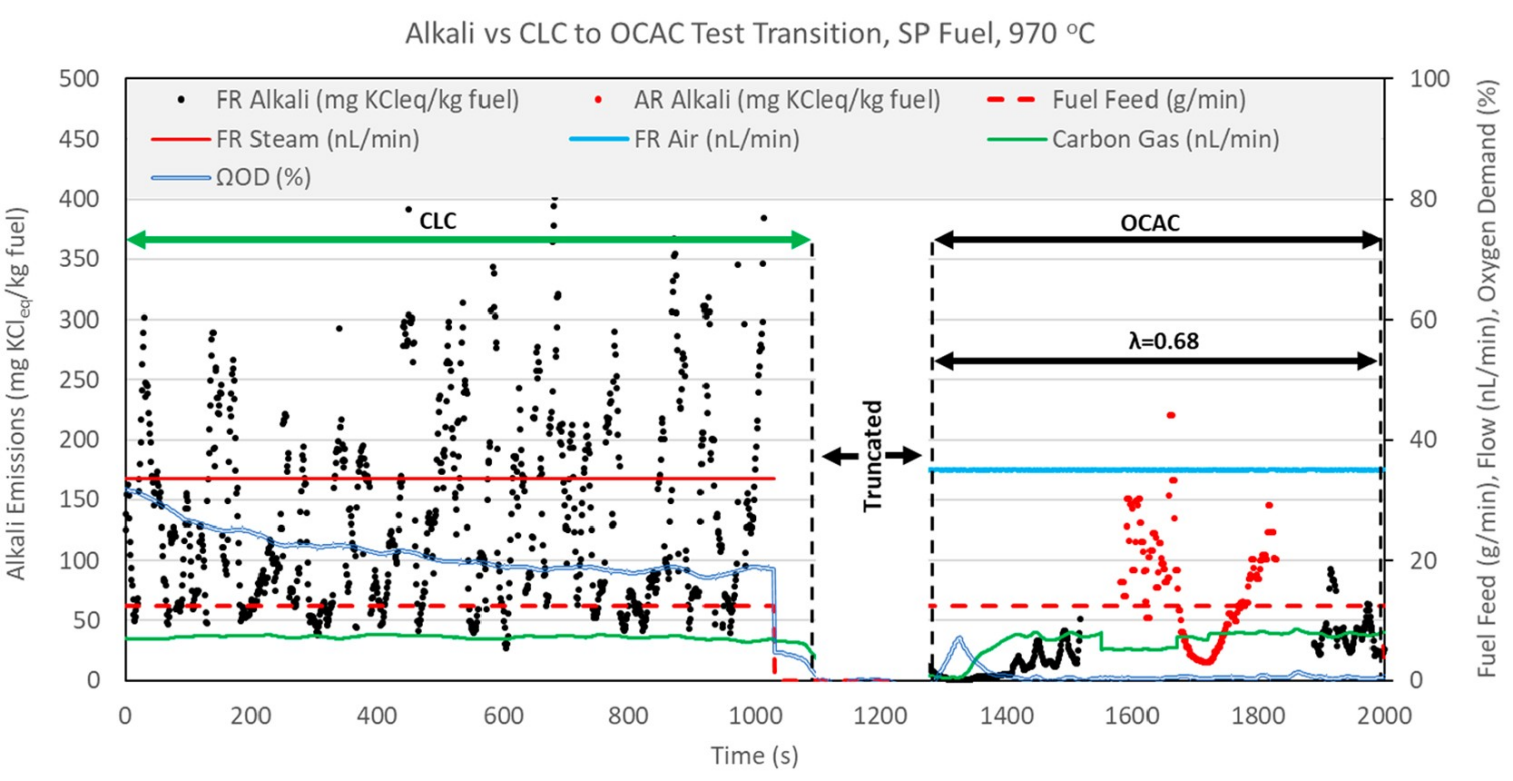
CLC versus OCAC alkali emissions for SP fuel operation at 970 °C.

Valuable insight into alkali release in a chemical looping system can be gained by operating the fuel reactor in OCAC mode. OCAC operation was achieved by switching FR fluidization from steam to air while maintaining fuel feed to the FR and air flow to the AR. OCAC operation was carried out following CLC operation for each of the biomass fuels. The AR flow rate, which controls OC circulation, and the fuel feed rate in OCAC operation were set to the same values as in the preceding CLC operation. Figures 12–14 show the measured alkali emissions and relevant process parameters for the CLC–OCAC test pairs. It should be noted that CLC operation for BP and PFR fuels was conducted immediately prior to OCAC operation, but for the SP fuel, several hours of CLC and CLG operations at varying process settings were conducted prior to OCAC operation. The data in Figures 12–14 have been truncated to remove data irrelevant to the CLC versus OCAC comparison.

Figures 12–14 show that FR alkali emissions decrease in OCAC operation when compared to CLC operation with the same fuel feed rate and solid circulation rate. Unfortunately, the OCAC operation shown in the figures does not represent ideal OCAC conditions because the air ratio λ (molar ratio of actual air to stoichiometric air) maintained in the FR system was below 1 (λ < 1). This means that the supply of oxygen from the air used to fluidize the FR was sub-stoichiometric. However, it is important to realize that because OC circulation was maintained at the same level in these two test types, in OCAC operation, oxygen is also supplied by the bed material at approximately the same rate as in the preceding CLC operation. Figures 12–14 include the calculated oxygen demand for the CLC and OCAC tests. For each fuel, the oxygen demand in OCAC operation is much lower than in the preceding CLC operation, indicating a higher degree of fuel conversion in OCAC versus CLC operation. Thus, even at λ < 1 conditions, the overall char conversion in OCAC should be similar or higher than in CLC operation. Figures 12 and 13 show that the flow of carbon gas is slightly higher in OCAC versus CLC operation for BP and PFR fuels. This indicates that, even though fluidizing air is supplied in less than the stoichiometric amount, char conversion in OCAC is likely higher than in CLC operation. For the SP fuel test, a comparison of carbon gas flow indicates that char conversion is similar in CLC and OCAC cases. This is also confirmed by the estimate of char conversion presented in Table 7. In summation, in comparison of CLC versus OCAC operation at the same reactor temperatures, OCAC operation results in a significant drop in FR alkali emissions, despite similar or higher char conversion.

### 4.5 Effect of the Steam Concentration on Alkali Emissions

**Figure 15:**
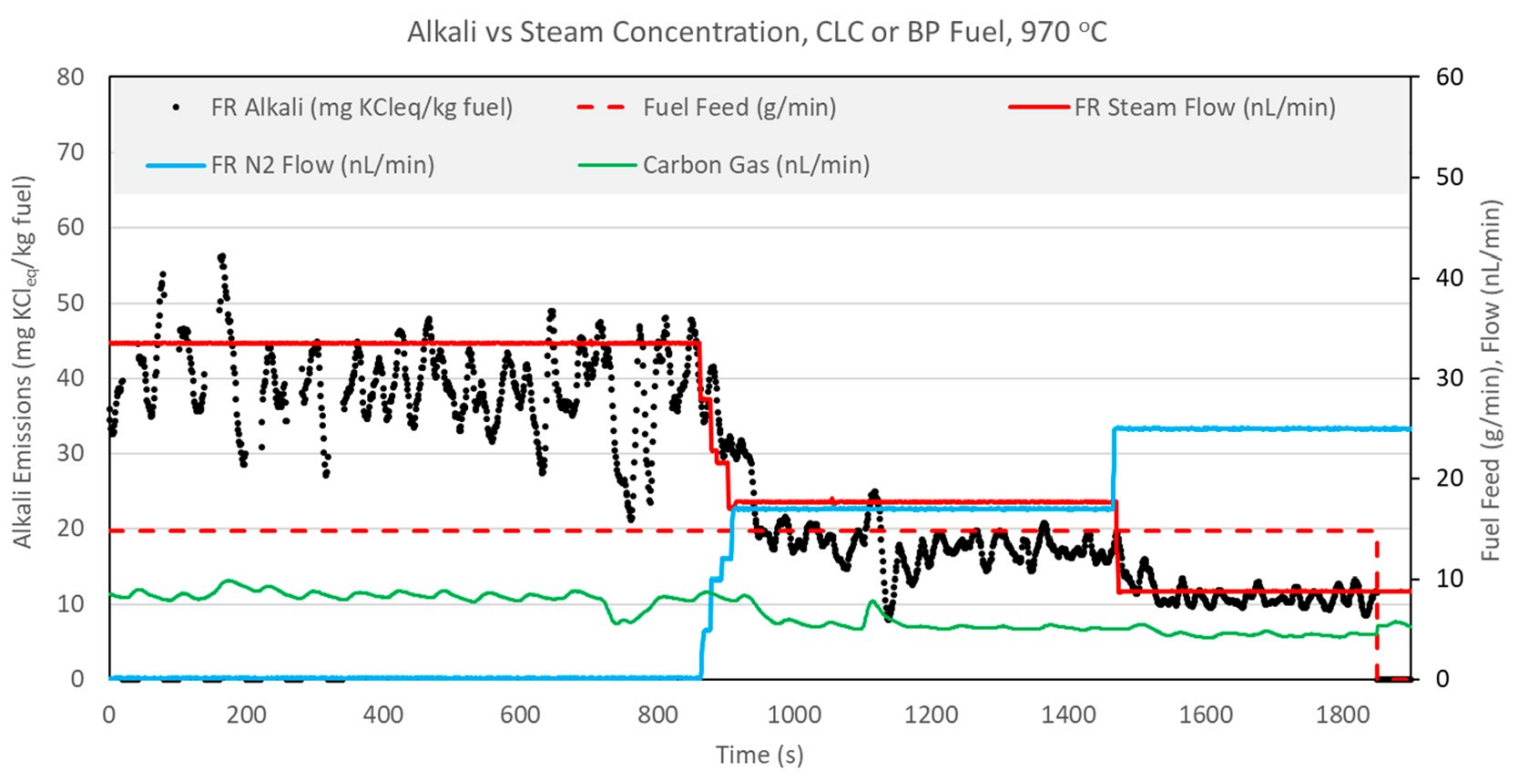
Effect of steam reduction on FR alkali emissions during CLC with BP fuel operation at 970 °C.

**Figure 16:**
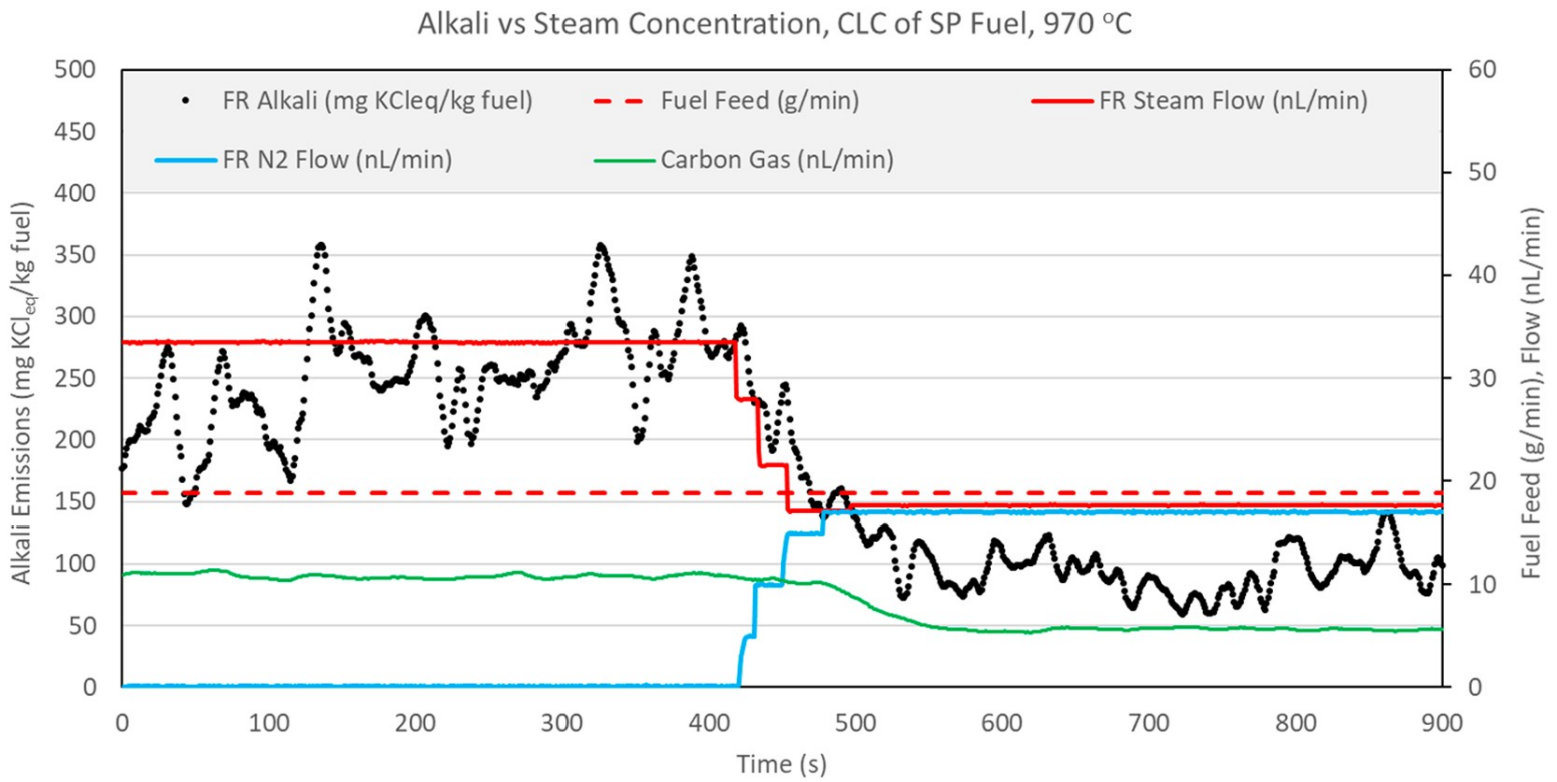
Effect of steam reduction on FR alkali emissions during CLC with SP fuel operation at 970 °C.

The finding that OCAC operation results in lower FR alkali release is consistent with results from a previous CLC campaign conducted on a D-CFB CLC pilot.^32^ In the D-CFB campaign, CLC and OCAC operations were also compared but OCAC was run with λ > 1, assuring high conversion of char. Like in the present study, FR alkali emissions in OCAC were much lower than those in CLC operation. The analysis of the D-CFB campaign results led to a hypothesis that the presence of steam in CLC is responsible for higher gas-phase alkali emissions in CLC versus a comparable OCAC operation.^32^ This hypothesis was further explored in the present study by conducting tests with stepwise reduction of the steam flow rate during CLC operation with BP and SP fuels. To keep the fluidization regime in the FR constant, reduction in steam flow was compensated by the addition of nitrogen to the FR wind box. Other parameters, such as the AR flow rate (controls the OC circulation), reactor temperature, and fuel feed rate, were kept constant. FR alkali emissions along with relevant process parameters for the steam reduction tests are shown in Figures 15 and 16.

**Figure 17:**
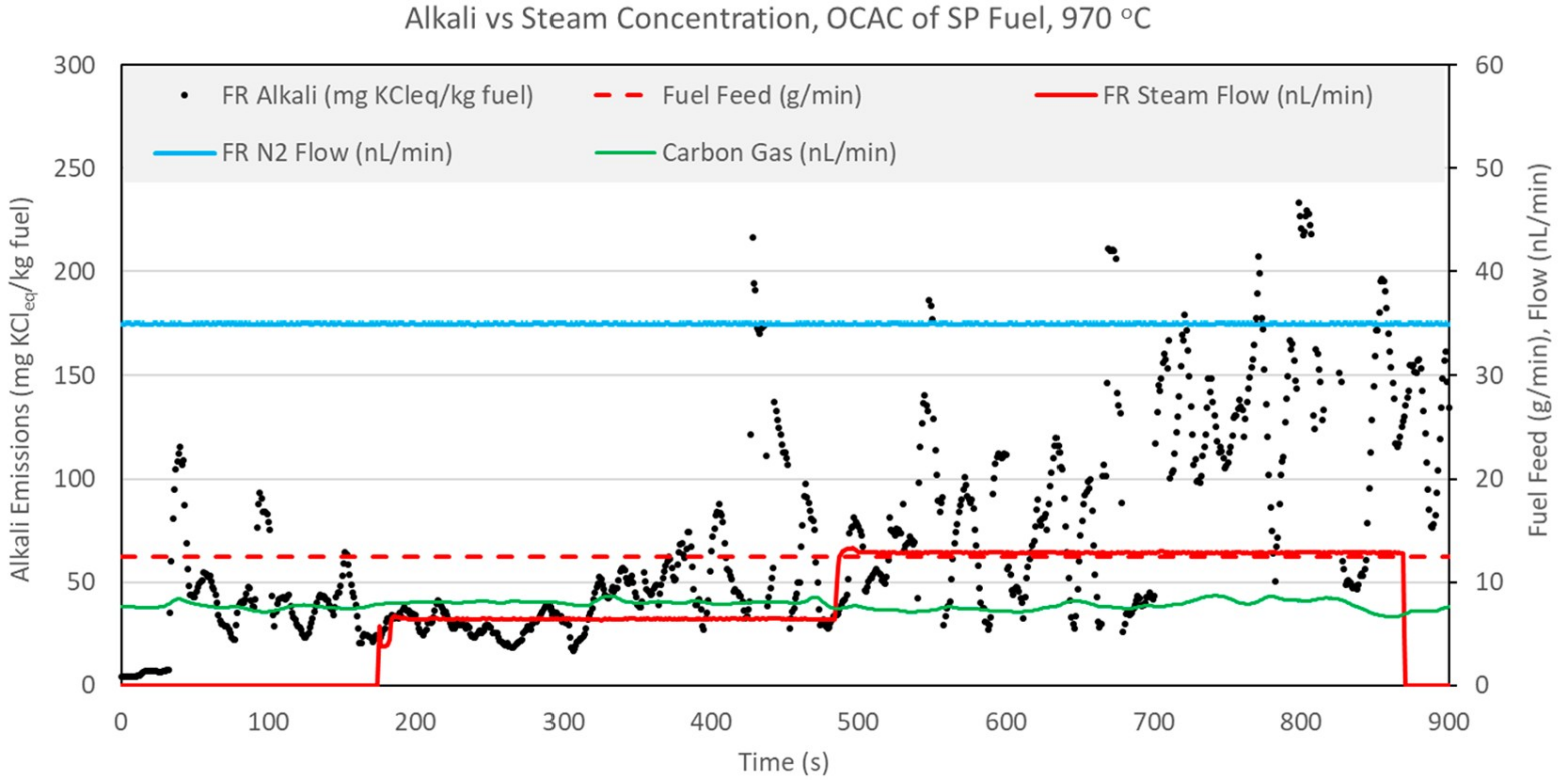
Effect of increased steam addition on FR alkali emissions during OCAC with SP fuel operation at 970 °C.

Figures 15 and 16 show that stepwise reduction in the steam flow rate during CLC operation results in a proportional reduction of FR alkali emissions. One possible explanation is that steam affects the rate of char conversion and, thus, influences the release of alkalis, which are known to be primarily concentrated in the char after char devolatilization. Evidence of this is provided in Figures 15 and 16 by the carbon gas flow rate parameter, which clearly decreases with steam reduction for both BP and SP fuels. Thus, it seems like a decrease in steam causes both lower char conversion and lower alkali release to the gas phase. However, it is not clear whether the reduction of char conversion and alkali emissions are interdependent or merely coincide. As discussed earlier, switching CLC operation to OCAC operation resulted in lower alkali release, despite an equivalent or higher char conversion in OCAC versus CLC. To further investigate a possible interdependency of alkali release and char conversion, another steam test was conducted during OCAC operation with SP fuel. The FR alkali emission measurements and relevant process parameters are presented in Figure 17.

In the test shown in Figure 17, steam is incrementally introduced during OCAC operation with SP fuel. The FR alkali concentration increases in response to the introduction of steam. The carbon gas flow, however, stays relatively constant, indicating a constant char conversion. The test results presented in Figure 17 suggest that the effect of steam on char conversion and alkali release can be decoupled. Steam seems to have a direct impact on alkali release.

As mentioned prior, the main release of alkalis to the gas phase occurs via evaporation of KCl(s,l) from the char. Dayton et al. showed that this process is influenced by the presence of steam, such that some KCl(g) reacts with steam to produce KOH(g) and HCl(g). This effect was found to be minor because KCl(g) is thermodynamically favored over KOH(g).^45^ In the present SID measurements, a shift from KCl(g) to KOH(g) would result in a lower signal as a result of lower sensitivity of the instrument to KOH(g). However, the SID signal increases with increased steam addition. Moreover, reforming of KCl(g) to KOH(g) would not change the net amount of gas-phase K that originated from evaporation of KCl(s,l) in the char.

As mentioned previously, beyond KCl evaporation, at the temperature levels of the current experiments, release of K from char to the gas phase is assumed to occur from the decomposition of alkali salts to the gas phase. For fuels with the K content exceeding the Cl content on an atomic basis, K_2_CO_3_ is known to be a likely form of K that is either present in precipitated form in virgin dry fuel^46,47^ or forms in the char during the fuel devolatilization step of fuel conversion.^19,20,48^ For the fuels used in the current experiments, a rough estimate for the potential of K to occur as K_2_CO_3_ can be made with the assumption that K prefers association with Cl and S to form KCl and K_2_SO_4_ prior to forming K_2_CO_3_.^20^ With this assumption, the atomic ratio of K/(Cl + 0.5S) > 1, calculated from the elemental analysis of the fuel, would suggest that there is a strong possibility for K occurrence as K_2_CO_3_. For the current experiments, the K/(Cl + 0.5S) atomic ratios of the fuels are 2.5, 8.8, and 4.4 for BP, PFR, and SP fuels, respectively (see Table 3). Thus, the presence of a significant portion of fuel K as K_2_CO_3_ in the char is highly probable. In a dry atmosphere, K_2_CO_3_(s,l) in the char will decompose to K(g) and CO_2_(g) during the char conversion process. This process will compete with a kinetically limited process of association of K with Si to form stable condensed phase silicates. The decomposition of K_2_CO_3_ in dry conditions is known to be a relatively slow process. However, if the atmosphere surrounding the char is moist, such as the steam-rich conditions in CLC, K_2_CO_3_ decomposition proceeds through a reaction of K_2_CO_3_(s,l) with steam to form KOH(g) and CO(g). At a given temperature, the wet decomposition path has been shown to be orders of magnitude faster than the dry decomposition process.^19,20,48,49^ In competition with K retention through silicate formation, faster decomposition of K_2_CO_3_ will result in a higher net release of K to the gas phase. Furthermore, the onset of the wet decomposition process was shown to occur at temperatures as low as 700 °C versus the dry path only becoming significant at temperatures over 900 °C.^20^ Given the evidence from the literature and the clear response of alkali emissions to steam addition demonstrated in Figures 15 and 16, we conclude that the steam addition increases net gas-phase release of K through steam-accelerated decomposition of K_2_CO_3_ during char conversion.

### 4.6 Retention of K by the OC and Overall System K Balance

**Figure 18:**
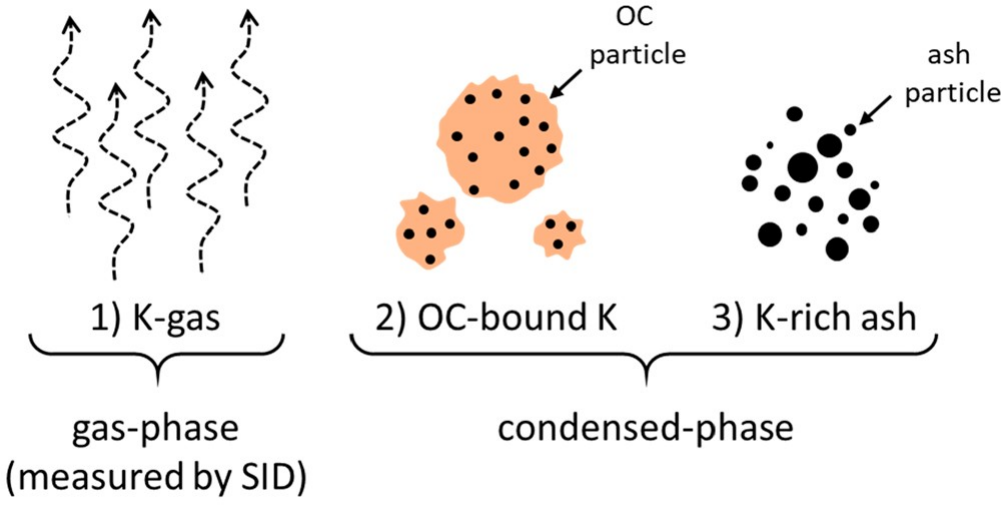
Forms of alkalis present in the reactor system.

At process conditions, alkalis in the CLC system can be present as condensed-phase and gas-phase species. Figure 18 presents a graphic representation of the possible alkali forms. Gas-phase alkalis are measured by the SID. Alkalis in the condensed form can occur in two different forms. One possibility is that alkalis bind to the oxygen carrier material through interaction of the OC material with gaseous alkalis or fuel ash. Another possibility is for alkalis to be present in a condensed form in fuel ash particles that are separate from the main OC fraction. In the determination of how these forms of alkalis are distributed in the system, solid sample analysis for the K content may offer some insights. K concentrations found in the different solid samples are shown in Table 8 for samples taken at approximately the same time and, thus, the same exposure to K.

**Table 8:** K Concentration in Solid Samples

day	fuel	temperature set point (°C)	bed material (mg of K/kg)	chimney deposits (mg of K/kg)	AR filter material (mg of K/kg)
1	BP	870	360	450	2100
2	BP	970	470	460	1400
3	PFR	870	470	610	1100
		920	600	650	n/a
		970	590	720	n/a
4	BP	870	840	910	1500
		920	860	n/a	1300
5	PFR	920	n/a	1300	1400
		970	n/a	1200	1200
6	SP	870	950	1300	n/a
		970	1300	1200	8600

Table 8 shows that the K concentration of the bed material increased over the duration of the campaign as the bed material was exposed to more fueled operation and fuels of increasing K content. K found in the bed material should be present in condensed form as either OC-bound K or K-rich ash. Selected bed material samples were analyzed by SEM. No clear separate ash particles were identified in the analysis, suggesting that most K held in the FR samples is in the OC-bound form. EDX of the particle cross sections was also performed. The low K content of the sample made interpretation of EDX results quite challenging. Nevertheless, elemental mapping of the particle cross sections suggested that K is finely distributed throughout the entire particle and is also present in a few locations in a more concentrated form that is associated with Al and Si. To confirm this association, FactSage thermodynamic modeling software was used to determine possible stable K compounds that can form from the major species found in LS slag OC (see composition in Table 1). The only stable condensed phase compound identified by FactSage was KAlO_2_. Most Si was predicted to be bound as Ca_2_SiO_4_ and Ca_3_MgSi_2_O_8_.

**Figure 19:**
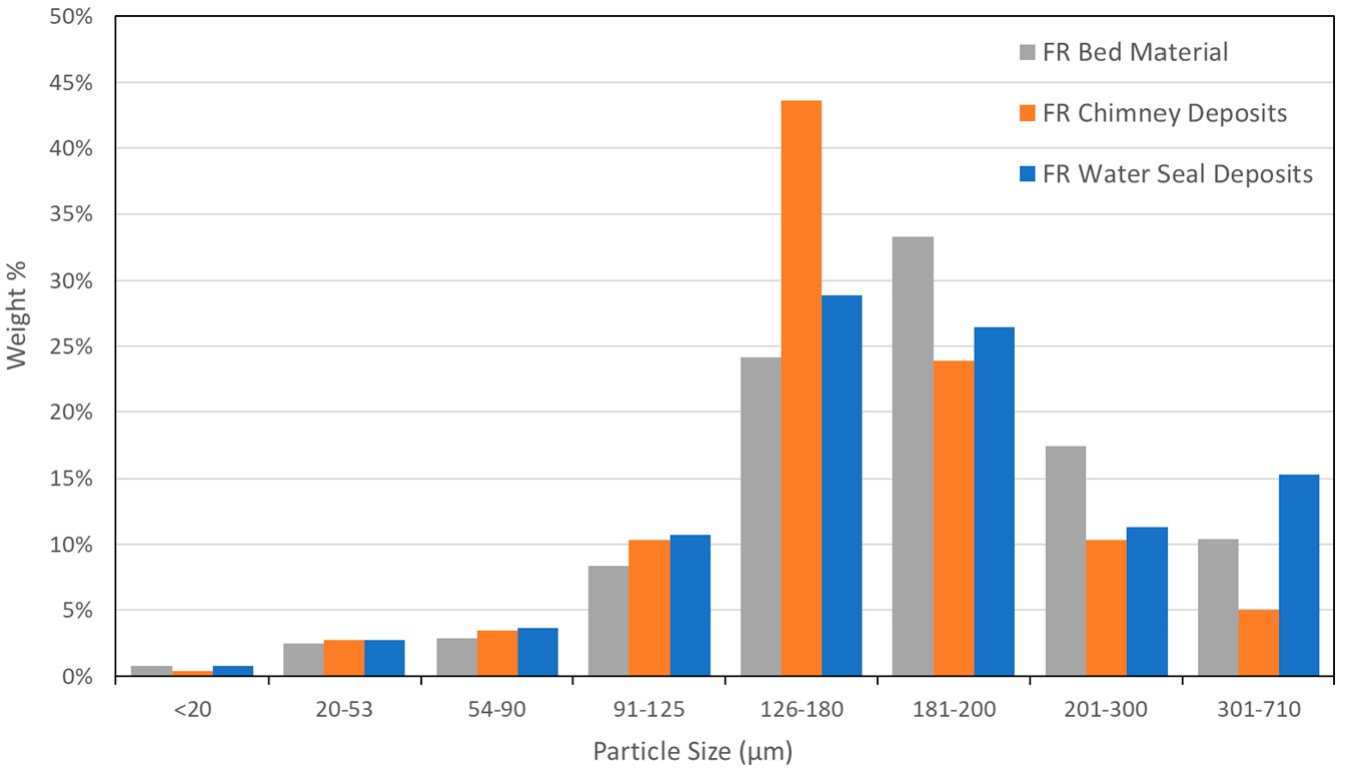
Particle size distribution for solid samples from day 5.

The K content of the FR chimney samples was found to be similar or slightly higher than that of the bed material samples. The particle size distribution was measured for a bed material sample, a FR chimney deposit sample, and a water seal deposit sample collected at the end of day 5 of operation. The particle size distributions, shown in Figure 19, show that the FR chimney deposits have a similar size distribution to the bed material samples. The similar size and K content suggest that chimney deposits are essentially elutriated bed material particles and do not contain a lot of fly ash. SEM/EDX of selected chimney deposit particle samples confirmed the absence of ash particles.

Figure 19 shows that the particle size distribution of the water seal deposit samples is also similar to that of the bed material. As mentioned prior, the K content of the water seal samples was not analyzed, because this material is continuously washed with water in the water seal. For the purposes of estimating the retention of K by the OC bed material, the water seal deposits were considered to be part of the OC fraction with a K concentration that is the same as that of the chimney deposits.

Analysis results presented in Table 8 show that AR filter particles had a K concentration that is significantly higher than that of the bed material. These particles were too fine and cohesive for PSD determination by sieving but were visually inspected with SEM and determined to have an approximate size of 5–10 μm. As a result of their smaller size and significantly higher K content, AR filter particles were classified as AR fly ash particles for the purpose of creating an overall system K balance. Using the methodology outlined in section 3.5, the percent retention of K by the OC and an overall K mass balance were developed for the CLC system. These results are presented in Table 9.

**Table 9:** CLC System Potassium Mass Balance

day	fuel	K in with fuel (mg of K)	percentage of fuel K retained by OC (%)	percentage of fuel K in AR gas phase (%)	percentage of fuel K in AR fly ash (%)	percentage of fuel K in FR gas phase (%)	unaccounted K (% of fuel K)
1	BP	2229	31 ± 254	1.4–4.7	3.2 ± 0.2	1.3–4.3	57 ± 254
2	BP	1343	180 ± 404	1.8–5.9	14 ± 1.6	5.1–17.2	–117 ± 408
3	PFR	12717	27 ± 55	0.2–0.6	3.8 ± 0.6	0.4–1.3	68 ± 56
4	BP	3146	89 ± 213	0.4–1.2	25 ± 2.9	1.4–4.6	–20 ± 216
5	PFR	11138	32 ± 64	0.1–0.2	3.3 ± 0.4	0.4–1.4	63 ± 64
6	SP	70950	33 ± 18	0.1–0.3	3.1 ± 0.3	0.6–1.9	62 ± 18

Results shown in Table 9 for retention of fuel K by the OC and the AR fly ash are based on the K concentration of solid samples measured with ICP–OES. In the conducted experiments, the relatively short fueled operating times meant that the OC inventory of the pilot system was exposed to relatively low amounts of K during each test day. As such, even if all fuel K is absorbed by the OC, the rise in the K concentration of the OC would be quite minor, especially for the low-K-content fuels. The uncertainty of the ICP–OES analysis of the K content was ±160 mg of K/kg of material. This level of measurement uncertainty and the low OC exposure to K resulted in a very high uncertainty in the estimates of K retention by the OC, as shown in column 4 of Table 9. The measurement uncertainty is only reasonable in the SP fuel case, where the K retention by the OC is estimated to be 33 ± 18 or 15–51%. Estimate uncertainty for PFR fuel operation is quite large but indicates that K retention by the OC was <82% on day 2 and <96% on day 5.

The uncertainty of the ICP–OES method is also reflected in the estimates of K retention by the AR fly ash. However, because significant K concentrations were measured in the AR filter sample material, the relative uncertainties of the estimated AR fly ash K retention are quite reasonable. As such, results in Table 9 suggest that K retention by the AR fly ash accounts for approximately 3–4% of fuel K in all tests with SP and PFR fuels and in BP fuel operation on day 1. For BP fuel operation on days 2 and 4, the retention of K by the AR fly ash is estimated to be 14 and 25%, respectively. Unfortunately, the reason for the difference in results for BP operation could not be established.

Results for SP fuel tests presented in Table 9 give the most complete and least uncertain overview of the alkali partitioning in the CLC pilot. SP mass balance results suggest that approximately 15–55% of fuel K is absorbed by the OC material, around 2.8–3.4% of fuel K is captured in the AR fly ash, and up to 2.2% of fuel K is detected in the AR and FR flue gases. The balance, 40–80% of fuel K, remains unaccounted. The fact that the SID measurements account for gas-phase K suggests that unaccounted K would have to be present in the condensed phase at process conditions. Of the two forms of condensed-phase K, the OC-bound form is accounted for by the bed material, the chimney deposits, and the water seal deposits. With the AR fly ash fraction also taken into account, it is likely that unaccounted K exists in the FR as K-rich fly ash with a particle size that is small enough to avoid deposition on the FR chimney walls and settling out in the FR water seal. This FR fly ash can possibly consist of individual fine fuel ash particles or as combination of fuel ash and LD slag OC fines. Although the presence of this K-rich FR fly ash could not be validated directly in these experiments, the AR filter deposits resemble this type of particle. Considering that AR fly ash particles can only originate from the FR, where fuel is converted, suggests that AR fly ash particles are a small fraction of the fly ash formed in the FR, which is carried over to the AR, while the bulk of the fly ash is stripped from the FR bed.

## 5 Conclusion

Alkali release in chemical looping combustion and gasification was investigated in a 10 kW_th_ pilot system. The pilot was operated with a LD slag (steel production slag) oxygen carrier and three different biomass fuels: BP, PFR, and SP. The experiments investigated how biomass alkalis are released and distributed in the two-reactor scheme of the CLC pilot and how the reactor temperature, mode of operation, and steam-rich environment of the fuel reactor affect the release of alkalis to the gas phase. Experiments were performed at three different temperatures (870, 920, and 970 °C) and in three different operation modes: CLC, CLG, and OCAC. Gas-phase alkali emissions were measured with a SID system.

Interpretation of the obtained experimental results was supported by concepts established in earlier investigations of alkali release in CLC and by literature on alkali release in thermal conversation of biomass. On those bases, it was concluded that, in chemical looping systems, the majority of alkali release to the gas phase occurs during the char conversion step of the fuel conversion process. Within the char conversion process, gas-phase alkali release occurs via evaporation of KCl and the decomposition of alkali salts to gaseous species. In the conducted experiments, the extent of alkali release to the gas phase was determined to be governed by three key parameters: extent of char conversion, reactor temperature, and steam concentration. From these, the extent of char conversion limits the overall time frame of the alkali release process, while the temperature and steam concentration affect the rates of the alkali release process within the time frame set by the extent of char conversion. The effect of each of these three variables was assessed systematically.

CLC and CLG tests performed at three different temperatures but fixed steam addition and char conversion showed that FR alkali emissions rise with the temperature. This effect was attributed to the fact that a higher temperature increases the vapor pressure of KCl and is known to enhance the decomposition of alkali salts to the gas phase. When looking at CLC versus CLG operation at the same temperature and steam concentration, FR gas-phase alkali release was found to be equivalent as a result of the equivalent extent of char conversion in CLC and CLG. Experiments comparing CLC to OCAC operation were conducted at a fixed temperature and similar extents of char conversion. In terms of gas-phase alkali release, the principle difference in these two modes is the presence of steam in CLC and absence of steam in OCAC. A comparison of FR alkali emissions in these two modes showed that the presence of steam in CLC increases gas-phase alkali emissions. The effect of steam was further explored in operational tests that show how alkali emissions respond to changes in the steam concentration. The tests showed that a decrease in the steam concentration in CLC operation results in lower FR alkali emissions but also a lower extent of char conversion. The decreased char conversion at the lower steam rate is explained by the fact that char conversion in CLC occurs via steam gasification of char. The step test in OCAC operation showed that FR alkali emissions rise with a higher steam concentration. This occurred even though char conversion was constant and mostly independent of steam in the OCAC case, because char is converted by char reacting with air in the OCAC case. This test confirms that the steam concentration has a direct effect on FR gas-phase alkali release. From these test results and a review of the fuel composition, it was concluded that a significant fraction of fuel alkalis are likely present in the char as K_2_CO_3_ and are more effectively released to the gas phase of the FR in the presence of steam by a reaction of K_2_CO_3_ with steam to yield KOH(g).

In the conducted experiments, AR gas-phase alkali emissions were found to be relatively constant and independent of the reactor temperature. Because significant char carryover was confirmed not to occur during the experiments, it was concluded that alkalis are carried over to the AR in OC-bound form or as ash particles. The exact pathway, however, could not be confirmed. In comparison of gas-phase alkali release in the FR versus the AR, the concentration of gas-phase alkalis in the AR was found to be lower than that in the FR. However, as a result of a higher gas flow rate of the AR, the net amount of gas-phase alkali release from the AR was equivalent to the FR for tests with BP fuel at 870 °C. For all other tests, the concentration and absolute release of gas-phase alkalis were higher in the FR versus the AR.

To supplement SID alkali measurements, the K content of various solid samples was collected throughout the experimental campaign. Analysis of the K content of the samples along with SID measurements was used to develop an overall K mass balance for the system. The short fuel operation times and large uncertainty in measuring the K concentration in the bed material samples resulted in very large uncertainties in mass balance results for BP and PFR fuel tests. Mass balance results for SP fuel tests were within a reasonable range of uncertainty and showed that LD slag OC absorbs approximately 15–51% of fuel K, gas-phase release accounts for up to 2.2% of fuel K, and up to 3.4% of fuel K is captured in the AR fly ash. The residual portion, 40–80% of fuel K, was determined to leave the FR in the form of FR fly ash.

## Nomenclature

AR: air reactor
BP: black pellets
BECCS: bioenergy with carbon capture and storage
CLC: chemical looping combustion
CLG: chemical looping gasification
CPC: condensation particle counter
EDX: energy-dispersive X-ray analysis
FR: fuel reactor
ICP–OES: inductively coupled plasma optical emission spectroscopy
MFC: mass flow controller
OC: oxygen carrier
OCAC: oxygen-carrier-aided combustion
PFR: pine forest residue
SEM: scanning electron microscopy
SID: surface ionization detector
SMPS: scanning mobility particle sizer
SP: straw pellets
Ω_OD_: oxygen demand (%)
Φ_o_: (moles of O_2_ to combust 1 kg of fuel)/(moles of C per kilogram of fuel)
